# USP28 controls SREBP2 and the mevalonate pathway to drive tumour growth in squamous cancer

**DOI:** 10.1038/s41418-023-01173-6

**Published:** 2023-05-18

**Authors:** Carina R. Maier, Oliver Hartmann, Cristian Prieto-Garcia, Kamal M. Al-Shami, Lisa Schlicker, Felix C. E. Vogel, Silke Haid, Kevin Klann, Viktoria Buck, Christian Münch, Werner Schmitz, Elias Einig, Bastian Krenz, Marco A. Calzado, Martin Eilers, Nikita Popov, Mathias T. Rosenfeldt, Markus E. Diefenbacher, Almut Schulze

**Affiliations:** 1 German Cancer Research Center, Division of Tumor Metabolism and Microenvironment, Im Neuenheimer Feld 581, 69120 Heidelberg, Germany; 2 Protein Stability and Cancer Group, Department of Biochemistry and Molecular Biology, Theodor-Boveri-Institute, Biocenter, Am Hubland, 97074 Würzburg, Germany; 3 Institute of Biochemistry II, Goethe University Frankfurt, Theodor-Stern-Kai 7, Haus 75, 60590 Frankfurt am Main, Germany; 4 Institute of Pathology, Julius Maximilians University and Comprehensive Cancer Center (CCC) Mainfranken, Josef-Schneider-Strasse 2, 97080 Würzburg, Germany; 5 Theodor Boveri Institute, Department of Biochemistry and Molecular Biology, Biocenter, University of Würzburg, 97074 Würzburg, Germany; 6 Internal Medicine VIII-Clinical Tumor Biology, University of Tübingen, Otfried-Müller-Straße 14, 72076 Tübingen, Germany; 7 Instituto Maimónides de Investigación Biomédica de Córdoba (IMIBIC), Córdoba, Spain; 8 Departamento de Biología Celular, Fisiología e Inmunología, Universidad de Córdoba, Córdoba, Spain; 9 Hospital Universitario Reina Sofía, Córdoba, Spain; 10 Present Address: Institute of Biochemistry II, Goethe University Frankfurt, Theodor-Stern-Kai 7, Haus 75, 60590 Frankfurt am Main, Germany

**Keywords:** Non-small-cell lung cancer, Deubiquitylating enzymes, Sterols, Gene regulation

## Abstract

SREBP2 is a master regulator of the mevalonate pathway (MVP), a biosynthetic process that drives the synthesis of dolichol, heme A, ubiquinone and cholesterol and also provides substrates for protein prenylation. Here, we identify SREBP2 as a novel substrate for USP28, a deubiquitinating enzyme that is frequently upregulated in squamous cancers. Our results show that silencing of USP28 reduces expression of MVP enzymes and lowers metabolic flux into this pathway. We also show that USP28 binds to mature SREBP2, leading to its deubiquitination and stabilisation. USP28 depletion rendered cancer cells highly sensitive to MVP inhibition by statins, which was rescued by the addition of geranyl-geranyl pyrophosphate. Analysis of human tissue microarrays revealed elevated expression of USP28, SREBP2 and MVP enzymes in lung squamous cell carcinoma (LSCC) compared to lung adenocarcinoma (LADC). Moreover, CRISPR/Cas-mediated deletion of SREBP2 selectively attenuated tumour growth in a KRas/p53/LKB1 mutant mouse model of lung cancer. Finally, we demonstrate that statins synergise with a dual USP28/25 inhibitor to reduce viability of SCC cells. Our findings suggest that combinatorial targeting of MVP and USP28 could be a therapeutic strategy for the treatment of squamous cell carcinomas.

## Introduction

The ubiquitin-proteasome system controls a wide range of cellular processes. Conjugation of the 76 amino acid polypeptide ubiquitin to target proteins is achieved through the sequential action of E1, E2 and E3 enzymes that first activate and then conjugate ubiquitin to the target protein. The complexity of the system is achieved by the large number of E3 enzymes (up to 1000) that differ in their molecular structure and show substantial substrate specificity [1, 2]. The ubiquitination process is counteracted by deubiquitinating enzymes (DUB), a family of over 100 isopeptidases, that hydrolyse the peptide bond between the C-terminus of ubiquitin and a substrate (e.g. another ubiquitin molecule) [3]. Ubiquitin-specific proteases (USP) make up almost half of the DUBs encoded by the human genome.

USP28 was shown to control the stability of a number of cancer-relevant proteins, most notably MYC [4]. However, USP28 also promotes tumour suppressive pathways, for example by deubiquitinating TP53, a function that is counteracted by nuclear caspase 8 during tumour relapse [5]. USP28 also modulates the DNA damage response (DDR) in cancer cells by deubiquitinating and stabilising the checkpoint kinase 2 (CHK2), the TP53-binding protein 1 (TP53BP1) and Claspin (CLSPN) [6, 7], thereby preventing apoptosis but establishing cell cycle arrest to facilitate DNA repair [8]. However, the exact function of USP28 in regulating DNA double-strand repair is still under investigation [9, 10].

Interestingly, USP28 counteracts the activity of the F-box and WD repeat domain containing protein 7 (FBXW7), a tumour suppressor protein that is part of the SCF (Skp1, Cullin-1, F-box) protein complex and facilitates the ubiquitination and destabilisation of MYC among other proteins. USP28 antagonises FBXW7, resulting in MYC stabilisation [11–13]. However, in some cell types, USP28 can also deubiquitinate and stabilise FBXW7 itself [14], leading to the downregulation of FBXW7 targets [15].

Recently, USP28 was shown to be upregulated in squamous cell carcinoma (SCC) where it leads to the deubiquitination and stabilisation of ΔNP63 [16]. Genetic deletion of USP28 via CRISPR/Cas9 blocked tumour formation in a mouse model of lung squamous cell carcinoma (LSCC) driven by mutant *Kras*^*G12D*^ in combination with deletion of *T*r*p53* and *Lkb1* [16]. Moreover, acute deletion of *Usp28* using a dual recombinase model resulted in regression of tumours formed by induction of mutant *Kras*^*G12D*^ in combination with *Fbw7* deletion [17]. Furthermore, pharmacologic inhibition of USP28 negatively affected LSCC tumour growth and was well tolerated in vivo [16, 17]. In addition, USP28 supports oncogenic transformation of respiratory cells and its inhibition synergises with therapeutic strategies targeting the EGFR/RAS/ERK pathway in transformed lung epithelial cells [18]. Therefore, understanding the molecular mechanisms by which USP28 supports tumour formation is vital for the rational targeting of LSCC.

The sterol regulatory element binding proteins (SREBP1a, SREBP1c and SREBP2, encoded by the *SREBF1* and *SREBF2* genes, respectively) are basic helix-loop-helix leucine zipper transcription factors that control the expression of genes involved in fatty acid and cholesterol biosynthesis [19]. While SREBP1a and SREBP1c show selectivity towards regulation of fatty acid biosynthesis, SREBP2 controls the expression of enzymes of the mevalonate pathway (MVP), which has multiple metabolic outputs that connect to biosynthetic and signalling processes in cancer, including providing substrates for the prenylation of small G proteins [20]. Key steps in the MVP are catalysed by the enzymes 3-Hydroxy-3-Methylglutaryl-CoA Synthase 1 (HMGCS1), 3-Hydroxy-3-Methylglutaryl-CoA Reductase (HMGCR) and Farnesyl-Diphosphate Farnesyltransferase 1 (FDFT1). SREBPs are expressed as inactive precursors that reside in the membrane of the endoplasmic reticulum (ER). While the precise mechanism of activation of SREBP1 is incompletely understood, both SREBP1 and SREBP2 are activated by proteolytic cleavage in a sterol-dependent manner [19]. Once nuclear, the N-terminal portion of the protein comprising the DNA binding and transactivation domains is subject to further regulation, including FBWX7-dependent ubiquitination [21, 22]. Interestingly, binding of FBWX7 to SREBP depends on phosphorylation of the conserved CDK2-phosphodegron (CPD) motif by glycogen synthase kinase-3β (GSK3B) [21]. Given the antagonism between FBWX7 and USP28 outlined above, it therefore seems possible that SREBPs could also be substrates for USP28-dependent deubiquitination and stabilisation.

Here, we report the identification of SREBP2 as a novel substrate of USP28 in squamous cancer cells. Depletion of USP28 resulted in the destabilisation of mature SREBP2, reduced expression of MVP enzymes and rendered cancer cells highly sensitive to MVP inhibition by statins, which was rescued by precursors of protein prenylation. We also found that USP28 and SREBP2 proteins are highly expressed in tissues from human LSCC patients compared to adenocarcinoma and that deletion of *Srebp2* reduced tumour formation in a KRas/p53/LKB1 mutant mouse model of lung cancer. Moreover, statins synergised with a dual USP28/25 inhibitor in reducing viability of human LSCC cells. Our findings suggest that USP28 targets SREBP2 to drive LSCC tumour growth.

## Material and methods

### Tissue culture and reagents

Cell lines were obtained from ATCC (American Type Culture Collection, Chicago, IL, USA) and used at low passage. Cells were cultured in DMEM (A431, U2OS, HEK293T) or RPMI 1640 (NCI-H520, LUDLU1, CALU-1, NCI-H522, NCI-H1299, A549) supplemented with 10% foetal calf serum (FCS), 2 mmol/L glutamine and 100 U/mL penicillin/streptomycin (all Sigma). Delipidated FCS was prepared as previously described [23]. Cells were grown at 37 °C in a humidified incubator at 5% CO_2_ and regularly tested for absence of mycoplasma. Cell-permeable cholesterol, Co-enzyme Q10, Geranylgeranyl pyrophosphate ammonium salt, (R)-Mevalonic acid-lithium salt, MG-132 and Simvastatin were from Sigma. Nucleosides (100×, EmbryoMax) were from Merck. AZ1 was from Selleckchem.

### Gene silencing and CRISPR/Cas9 knock-out

For inducible gene silencing, shRNA sequences targeting human *USP28* and *SREBF2* were designed using splashRNA (http://splashrna.mskcc.org) and cloned into LT3-GEPIR vector (Addgene #111177). Viral particles were produced in HEK293T cells and stable populations were selected using puromycin (2 µg/mL). For CRISPR/Cas9 mediated gene silencing, sgRNA sequences were designed using CHOPCHOP (https://chopchop.cbu.uib.no) and cloned into pX458 (Addgene #48138). U2OS cells were transfected and selected with puromycin and single clones were picked. Silencing and knockout efficiency was confirmed using qPCR and western blotting.

shRNA sequences

shUSP28 #1

TGCTGTTGACAGTGAGCGCACAAGAGATTAGAAATATAAATAGTGAAGCCACAGATGTATTTATATTTCTAATCTCTTGTATGCCTACTGCCTCGGA

shUSP28 #2

TGCTGTTGACAGTGAGCGATACAAGAGATTAGAAATATAATAGTGAAGCCACAGATGTATTATATTTCTAATCTCTTGTAGTGCCTACTGCCTCGGA

shSREBF2 #1

TGCTGTTGACAGTGAGCGCTCTGTATATATTTAAACCTAATAGTGAAGCCACAGATGTATTAGGTTTAAATATATACAGATTGCCTACTGCCTCGGA

shSREBF2 #2

TGCTGTTGACAGTGAGCGAAAGGCCATTGATTACATCAAATAGTGAAGCCACAGATGTATTTGATGTAATCAATGGCCTTCTGCCTACTGCCTCGGA

CRISPR guide RNA sequences

sgCtrl fwd humanCACCGCGAGGTATTCGGCTCCGCG

sgUSP28_1 fwd humanCACCG GAGTTGATGGTTGGCCAGTT

sgUSP28_2 fwd humanCACCG ACCCCAATCCCAATGACTGG

sgSREBF2_#1 fwd mouseCACCGCTTCAGCGTGGTCAACACAA

sgSREBF2_#2 fwd mouseCACCGAGCGACCGTCTGTACCGTGG

### Proteomics analysis

Whole cell proteome analysis of A431 cells expressing shRNAs targeting USP28 or non-targeting controls and treated with 1 µg/mL doxycycline for 72 h has been described previously [10, 18]. Pathway analysis of USP28 regulated proteins (FDR *p*-value ≤ 0.05) was performed with the PANTHER tool using the statistical overrepresentation test and PANTHER pathways with default settings [19].

### RNAseq and data analysis

RNA was extracted using RNeasy columns (Qiagen). mRNA was isolated using NEBNext^®^ Poly(A) mRNA Magnetic Isolation Module and library preparation was performed with NEBNext® Ultra™ RNA Library Prep Kit for Illumina following the manufacturer’s instructions. Libraries were size-selected using Agencourt AMPure XP Beads (Beckman Coulter) followed by amplification with 12 PCR cycles. Library quantification and size determination was performed with an Experion system (Bio-Rad) and libraries were sequenced with NextSeq500 (Illumina) at DKFZ Genomics Core facility. Reads were aligned to the human genome (hg19) with TopHat2, Bowtie v0.12.8 using default parameters. Mapped reads per gene (EnsEMBL GRCh37, release 74) were counted using the “summarizeOverlaps” function in the GenomicAlignments R package, non-expressed genes were removed (mean read count per gene over all samples >1) and TMM normalized with EdgeR. Gene set enrichment analyses were performed with the C2 and Hallmark collection from the MSigDB v5.2 with default parameters and 1000 permutations. Principle component analysis (PCA) was performed with the prcomp function from R after centering sequencing depth-normalized expression values of all expressed genes (*n* = 20,434). RNAseq data are available at GEO (GSE204703).

### Detection of cholesterol and CoQ10 by LC-MS

Cells were incubated in DMEM medium without glucose (Sigma, D5030) supplemented with 25 mM [U^13^C]-glucose (Cambridge Isotope Laboratories, Inc.). After 48 h, cells were washed with 0.5 ml cold ammonium acetate (154 mM), snap-frozen in liquid nitrogen and scraped off in 0.5 mL ice-cold MeOH/H2O (80/20 v/v). The suspension was transferred to a glass tube and another 0.5 ml ice-cold MeOH/H2O (80/20 v/v) were added. Internal standards 7-dehydrocholesterol-d7 (Sigma-Aldrich) and coenzyme Q9 (Sigma-Aldrich) were used in the final concentrations of 2 and 0.24 µmol/l, respectively. After addition of 120 µl 0.2 M HCl, 360 µl chloroform, 400 µl chloroform and 400 µl of water with vigorous mixing between the pipetting steps, samples were centrifuged at 3,000 *g* for 10 min. 700 µl of the lower phase were collected and taken to dryness under a stream of nitrogen gas at 40 °C. The resulting residues were dissolved in 50 µl isopropanol and taken for LC-MS analysis.

For LC-MS analysis of cholesterol and ubiquinone, 3 µl of sample was applied to a C8 column (Accucore C8, 2.6 µm particles, 50 × 2.1 mm, Thermo Scientific). Mobile phase buffer A consisted of 0.1% formic acid in CH_3_CN/H_2_O (10/90, v/v) and mobile phase buffer B consisted of 0.1% formic acid in CH_3_CN/iPrOH/H_2_O (45/45/10, v/v/v). After sample application at 40 °C, the LC gradient program was 20% solvent B for 2 min, followed by a linear increase to 99.5% solvent B within 5 min, then maintaining 99.5% solvent B for 28 min, then returning to 20% B within 1 min. The column was equilibrated at 20% B for 5 min prior every injection. The flow rate was maintained at 350 µl/min. The eluent was directed to the ESI source of the MS instrument from 4.0 min to 35.0 min after sample injection. MS analysis was performed on a Q-Exactive plus mass spectrometer (Thermo Scientific). For the detection of cholesterol, the following scan and HESI source parameters were applied in positive mode: 350–400 m/z; resolution: 70,000; AGC-Target 3E6; maximum injection time: 200 ms; sheath gas: 30; auxiliary gas: 5; aux gas heater temperature: 350 °C; spray voltage: 3.2 kV; capillary temperature: 320 °C, S-lens RF level: 55.0. For the detection of coenzyme Q10, the following scan and HESI source parameters were applied in polarity switching mode: 200–1600 m/z; resolution: 70,000; AGC-Target 1E6; maximum injection time: 50 ms; sheath gas: 30; auxiliary gas: 5; aux gas heater temperature: 350 °C; spray voltage: 3.2 kV; capillary temperature: 320 °C, S-lens RF level: 55.0. Signal determination and quantitation was performed using El-Maven Software Version 0.12.0 (https://elucidata.io/el-maven/) and natural abundance correction was performed using FluxFix (http://fluxfix.science/).

### Immunofluorescence

Cells were grown on glass slides (Ibidi), washed with PBS and fixed with 3.7 % PFA for 10 min. Cells were permeabilized with 0.2 % Triton X-100 for 10 min, washed with PBS and blocked with 3 % BSA in PBS for 30 min. Primary antibodies were diluted in 1 % BSA in PBS and incubated overnight at 4 °C. Cells were washed twice with PBS and incubated with fluorescently labelled secondary antibodies (Alexa488 and Alexa633, Invitrogen) for 1 h in the dark. Finally, cells were washed three times with PBS and mounted with mounting medium containing DAPI (Duolink®, Sigma). Concentration of primary antibodies was experimentally determined (1:100–1:50), secondary antibodies were used in a concentration of 1:200.

### Transient transfection

Cells were transfected using Polyethylenimine (PEI, branched) (Sigma). Plasmid-DNA and PEI were separately prepared in Optimem in DNA:PEI ratio of 1:2. Both solutions were incubated for 5 min and afterwards mixed and incubated for 20 min at RT. Cells were washed once with PBS and medium was changed to transfection medium (DMEM, 2% FCS). DNA-PEI solution was added dropwise to the cells and incubated up to 24 h.

### Cell lysis, cell fractionation and western blotting

Cells were lysed in RIPA buffer (150 mM NaCl, 50 mM Tris pH 8.0, 1% (v/v) NP-40, 0.5% (w/v) sodium deoxycholate, 0.1% (w/v) SDS) with protease and phosphatase inhibitors for 30 min and cleared by centrifugation. Proteins were quantified using BCA (Thermo Scientific). Cell fractionation as performed using NE-PER™ Nuclear and Cytoplasmic Extraction kit (Thermo Scientific). Proteins were separated on SDS-PAGE and blotted onto PVDF membrane (Immobilon), treated with blocking solution (Thermo Scientific) and incubated with primary and secondary antibodies. Signals were detected on an Odyssey scanner (LI-COR) or ChemiDoc (BioRad). Antibodies used were: anti-SREBP2 (R&D, AF7119), anti-USP28 (Sigma, HPA006778), anti-USP25 (Sigma, HPA018297), anti-HMGCS1 (Abcam, ab155787), anti-calreticulin (Stressgen, SPA-600), anti-Lamin A/C (Proteintech, 10298-1), anti-ubiquitinated proteins (EMD Millipore, FK2 04-263), anti-actin (Sigma, A3854), anti-vinculin (Sigma, V9131). Fluorescent secondary antibodies were from LI-COR and BioRad. HR-coupled antibodies were from GE Healthcare.

### Proximity ligation assay (PLA)

Cells were grown on chambered coverslips (Ibidi), fixed, permeabilised and PLA was carried out using the Duolink kit (Sigma) according to manufacturer’s instructions. Slides were mounted with in situ Mounting Medium (Sigma) containing DAPI. Signals were detected using a confocal SP8 microscope (Leica).

### Immunoprecipitation (IP)

Cells were harvested by scraping, dithiobis[succinimidylpropionate] (DSP, Sigma) was added in a final concentration of 0.8 mM in PBS and incubated for 30 min. Pellets were lysed in fractionation buffer (20 mM HEPES pH 7.4, 10 mM KCl, 2 mM MgCl_2_, 1 mM EDTA, 1 mM EGTA) supplemented with protease inhibitors for 30 min, passed through a 27-gauge needle, incubated for 20 min and cleared by centrifugation. Pellets were washed and extracted in PBS with 0.1% SDS. Genomic DNA was sheared by passing through a 22-gauge needle. SREBP2-enriched fraction was cleared by centrifugation, protein concentration was determined by BCA. For direct IP, cells were lysed in RIPA buffer. Immunoprecipitation was performed using 0.75 mg of Dynabeads™ Protein A/G (Invitrogen), 5 μg of anti-SREBP2 (R&D, AF7119) or 5 µg of anti-USP28 (Sigma, HPA006778) and 400 μg (U2OS) or 1 mg (A431) of protein lysate. Goat or rabbit IgG was used as a control.

### Ni-NTA pull-down assay

HEK293T cells were transfected with plasmids encoding 6His-tagged K48-only ubiquitin, HA-tagged mSREBP2 and USP28-WT or USP28-CA using the Polyjet reagent (Signagen). 48 h after transfection, cells were lysed in Urea buffer (8 M Urea, 1%TX-100, 300 mM NaCl, 25 mM Imidazole) in PBS and briefly sonicated. Cleared lysates were incubated with Ni-NTA beads overnight to capture ubiquitinated proteins. Beads were washed three times with lysis buffer and precipitated proteins were denatured in sample buffer for separation on SDS-PAGE.

### Colony formation and cell viability using crystal violet staining

For colony formation experiments, cells were seeded in 6 cm plates at very low density and treated with doxycycline or solvent as indicated. For cell viability assay, cells were seeded in 96 well plates and treated as indicated. After incubation, cells were washed with PBS and fixed for 10 min in 3.7% paraformaldehyde (PFA). Cells were washed again and stained with 0.1% crystal violet solution for 1 h. Plates were rinsed in water, dried and extracted using 10% acetic acid. Absorbance was measured at 550 nm.

### Analysis of Cancer Genome Atlas (TCGA) data and survival analysis

Raw gene expression data of lung adenocarcinoma (LADC) and lung squamous carcinoma (LSCC) were extracted from TCGA datasets (firehose legacy) using cBioPortal (http://cbioportal.org). mRNA expression data (RNAseq V2 RSEM) were log2 transformed and compared using a non-parametric two-tailed Mann-Whitney test. Correlation analyses were performed using GEPIA2 [24]. Kaplan–Meier plots and survival analyses were performed using the KM plotter tool [25].

### Human lung cancer tissue microarrays (TMA)

Human lung cancer samples were obtained from the Pathology Department at the University Hospital Würzburg (Germany) with informed consent from all patients. Experiments were in agreement with the principles set out in the WMA Declaration of Helsinki and the Department of Health and Human Services Belmont Report. Samples were approved under Ethics Approval 17/01/2006 (University Hospital Würzburg). Additional human samples were obtained from the Instituto Maimónides de Investigación Biomédica de Córdoba (IMIBIC), Córdoba, Spain after informed consent was given approved under ethical approval licences Decret 439/2010 (Hospital Universitario Reina Sofia). TMAs were prepared as previously described [26]. In brief, paraffin moulds were cast using an Arraymold Kit (IHC World, Kit D, IW-115, core diameter 2 mm, 36 cores). Human samples were cut and stained using haematoxylin and eosin and digitalized using a 3D Histech slide scanner (panoramic FLASH). Tumour and non-transformed tissues were identified, manually ‘punched’ and transferred. Upon completion, 3-µm thick sections were cut using a microtome and processed.

### Immunohistochemistry

Paraffin-embedded sections of human and murine samples were cut into 4 µm sections with a microtome (Leica). Slides were de-paraffinized and rehydrated using the following protocol: 3 × 5 min in Xylene, 2 × 2 min in ethanol (100%), 2 × 2 min in ethanol (95%), 2 × 2 min in ethanol (70%), 2 min in ethanol (50%) and 5 min in H_2_O. Antigen retrieval was performed with 10 mM sodium citrate buffer (pH 6.0) in a microwave oven at 800 W, 650 W and 360 W for 5 min each. Samples were permeabilised with TBS 0.1 % Triton X-100 for 10 min and blocked with TBS containing 3 % H_2_O_2_ for 10 min. Human samples were stained with anti-SREBF2 (R&D, MAB7119), anti-USP28 (Sigma, HPA006778), anti-SREBF1 (PTG, 14088-1-AP) and anti-HMGCS1 (Abcam, ab155787). Murine samples were stained with anti-USP28 (Sigma, HPA006778), Nkx2-1 (TTF1; Santa Cruz sc-13040) anti-HMGCS1 (Abcam, ab155787), anti-SREBF2 (R&D, MAB7119), anti-Δnp63 (4A4) (Ventana, 05867061001) and anti-Cytokeratin 5 (Bimake, A5439). Slides were developed with DAB staining solution (SignalStainR DAB Substrate Kit, Cell Signaling 8059 S) and counterstained with haematoxylin (Sigma H3136). Slides were scanned at 40x resolution using a Pannoramic DESK II slide scanner (3D Histech) and analysed using QuPath (version 0.3.2).

### AAV production

To produce AAV particles, 5 million HHEK293T cells per dish were seeded in 15 cm plates and cultivated until a confluence of ~60–70 %. Cells were transfected with the pRepCap (pRC, Cell Biolabs Inc.), the cis-plasmid (pAAV) and the pAdDeltaF6 (Addgene #112867) in a 1:1:2 molar ratio by mixing the DNA in 2 ml DMEM (w/o FCS) and adding polyethylenimine (PEI, Polysciences (DNA:PEI ratio of 1:2). The mixture was incubated 15 min at room temperature and added dropwise to the plates. To harvest AAV particles, cells and supernatants were collected after 96 h and transferred into a 50 ml conical tube. At first, NaCl was added (final concentration 0.5 M) and slowly mixed for 1 h at 4 °C. Next, Chloroform was added (final concentration 10 %) and slowly mixed for 30 min at 4 °C. The suspension was centrifuged at 2000 × *g* for 30 min at 4 °C. Water phase was transferred into a new conical tube and PEG 8000 was added (final concentration 10 %) and mixed well. AAV was precipitated overnight at 4 °C. After the centrifugation at 2000 × *g* for 20 min at 4 °C the pellet was dissolved in AAV resuspension buffer (PBS + 0.001% pluronic F68 + 200 mM NaCl) (~100 µl/15 cm dish used) and protease inhibitor and DNase/RNase were added. Reaction was incubated for 2 h at 37 °C, chloroform in a ratio of 1:1 was added and samples were centrifuge at 12,000 × *g* for 5 min at 4 °C. The chloroform step was repeated and the water phase was collected. Virus titre was determined by quantitative PCR using primers against the ITR of the AAV as previously reported [27]. This procedure was previously described [28].

### Animal experiments

All in vivo experiments were approved by the Regierung Unterfranken and the ethics committee under the licence numbers 2532-2-362, 2532-2-367 and 2532-2-374. The mouse strain used was B6(C)-Gt(ROSA)26Sor^em1.1(CAG-cas9*,-EGFP)Rsky^/J (Jackson Laboratories, stock #028555). All animals were housed in standard cages in a pathogen-free facility on a 12 h light/dark cycle with ad libitum access to food and water. Animal health monitoring via sentinel animal screening is carried out in accordance with FELASA 2014 guidelines and conducted every 3 months. Adult mice (7–8 weeks old) were anaesthetized with isoflurane and intratracheally intubated with 60 µl AAV (1 × 10^11 PFU) diluted in PBS. Viruses were quantified using Coomassie staining protocol [29] and via qPCR (https://www.addgene.org/protocols/aav-titration-qpcr-using-sybr-green-technology/). At the indicated time point, animals were sacrificed by cervical dislocation and lungs were dissected and fixed using 4 % neutral buffered formalin (Sigma). Tumour burden was determined by calculating the percent tumour area relative to total lung area for each animal using QuPath 0.3.2 (https://qupath.github.io/). No animals were excluded from the analysis and tissues were analysed in a blinded manner.

### Statistical analysis

Statistical analysis was performed using R 3.10 (http://www.r-project.org) or Graphpad Prism 9 (Graphpad Software Inc.). Drug synergy was calculated using the synergyfinder tool (https://synergyfinder.fimm.fi) with ZIP model.

### Reporting summary

Further information on research design is available in the Nature Portfolio Reporting Summary linked to this article.

## Results

### USP28 regulates the mevalonate pathway

**Fig. 1:**
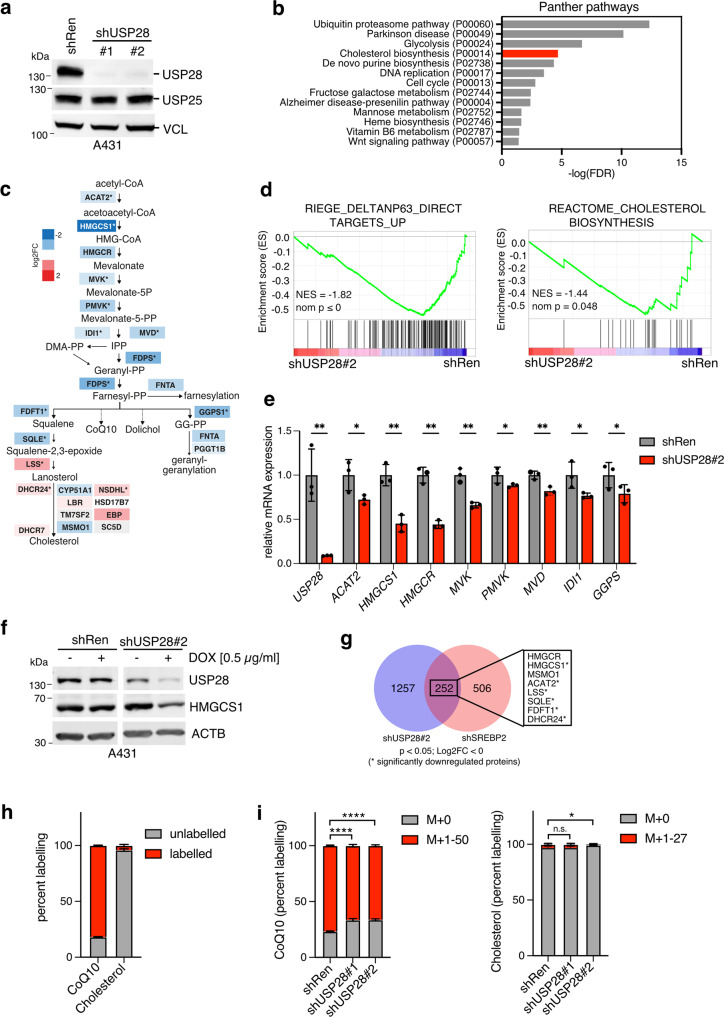
USP28 regulates the mevalonate pathway (MVP). **a** A431 cells expressing inducible shRNA sequences targeting USP28 (shUSP28#1 and shUSP28#2) or non-targeting control (shRen) were treated with 1 µg/ml of doxycycline (DOX) for 96 h. Protein lysates were analysed for expression of USP28 and USP25 by immunoblotting. Vinculin is shown as loading control. **b** Proteins differentially regulated by USP28 (shUSP28#1) silencing for 72 h in A431 cells [10] (FDR ≤ 0.05 compared to shRen, total of 2200 proteins) were subjected to pathway analysis using the PANTHER tool. **c** Differential expression of MVP proteins as determined by proteomics analysis. Pathway map is coloured by log2FC (^*^q-value ≤ 0.05, *n* = 3). **d** A431 cells expressing an inducible shRNA sequence targeting USP28 (shUSP28#2) or non-targeting control (shRen) were treated with 1 µg/ml of doxycycline (DOX) for 120 h. Changes in gene expression were analysed and enrichment plots for gene sets mapping to direct ΔNP63 targets and cholesterol biosynthesis are shown. **e** Validation of downregulation of mevalonate pathway genes following USP28 silencing using 1 µg/ml of doxycycline (DOX) for 96 h. Data are presented as mean ± SD of three independent experiments (^*^p < 0.05, ^****^p < 0.0001, unpaired two-tailed *t*-test with FDR). **f** A431 cells expressing an inducible shRNA sequence targeting USP28 (shUSP28#2) or non-targeting control (shRen) were treated with 0.5 µg/ml of doxycycline (DOX) for 96 h. Expression of USP28 and HMGCS1 were analysed by immunoblotting. Actin is shown as loading control. **g** Overlap between genes downregulated by silencing of USP28 and SREBP2 in A431 cells. Among the 252 overlapping genes are 8 mevalonate pathway enzymes (boxed) that also showed downregulation in the proteomics dataset. **h** A431 cells were incubated with medium containing 25 mM [U^13^C]-glucose for 48 h. Metabolites were extracted and analysed by LC-MS. Fractions of labelled and unlabelled ubiquinone (CoQ10) and cholesterol are shown. Data are presented as mean ± SD of three independent experiments. **i** A431 cells expressing inducible shRNA sequences targeting USP28 (shUSP28#1 and shUSP28#2) or non-targeting control (shRen) were treated with 1 µg/ml of doxycycline (DOX) for 120 h. During the last 48 h, cells were incubated with medium containing 25 mM [U^13^C]-glucose. Metabolites were extracted and analysed by LC-MS. Fractions of labelled and unlabelled ubiquinone (CoQ10) and cholesterol are shown. Data are presented as mean ± SD of three independent experiments (n.s. non-significant, ^*^p < 0.05, ^****^p < 0.0001, one-way ANOVA with post-hoc Dunnett’s test).

To study the consequences of USP28 depletion in squamous cell carcinoma, we generated A431 cells expressing doxycycline-inducible shRNA sequences targeting USP28 (#1 and #2) or non-targeting control (shRenilla). Doxycycline treatment for 96 h induced almost complete depletion of USP28 protein, while the related deubiquitinating enzyme USP25, which shows significant structural homology to USP28 [30, 31], was unaffected (Fig. 1a). Protein extracts of doxycycline-treated cells were submitted to quantitative LC-MS analysis [10] to identify global changes in cellular proteome. Pathway analysis (www.pantherdb.org) of proteins downregulated after USP28 depletion (FDR ≤ 0.05) revealed strong regulation of pathways linked to the ubiquitin-proteasome system, as well as DNA replication and cell cycle control (Fig. 1b). Interestingly, regulation of cholesterol biosynthesis was also strongly associated with USP28 silencing (Fig. 1b). Detailed analysis revealed significant downregulation of proteins mapping to the upper part of the cholesterol biosynthesis pathway, also known as the mevalonate pathway (MVP), which facilitates the synthesis of isoprenoids that can be used as substrates for protein prenylation [20]. Proteins of the lower part of cholesterol synthesis pathway downstream of SQLE were less strongly downregulated or even induced (Figs. 1c and S1a).

We next applied RNA sequencing (RNAseq) to establish the transcriptional response to *USP28* silencing in A431 cells. Gene set enrichment analysis revealed strong downregulation of direct ΔNP63 target genes in response to silencing of *USP28* (Fig. 1d), confirming our previous findings [16]. Furthermore, genes involved in cholesterol biosynthesis also showed significant downregulation (Fig. 1d, e), suggesting that the reduction in MVP pathway proteins is the consequence of altered transcriptional activity. Efficient downregulation of USP28 and HMGCS1 mRNA and protein was also achieved when cells were treated with 0.5 µg/ml of doxycycline, confirming specificity of the effect (Figs. 1f and S1b).

Transcription of MVP genes is primarily controlled by SREBP2, a basic helix-loop-helix transcription factor that is activated upon low intracellular sterol levels via regulated intramembrane processing [20]. We, therefore, investigated the transcriptional response to SREBF2 silencing in A431 cells. Silencing of *SREBF2* resulted in clear downregulation of cholesterol biosynthesis enzymes and also affected ΔNP63 target genes (Fig. S1c–g), suggesting that USP28 and SREBP2 functionally overlap. Moreover, we observed a substantial overlap between genes regulated by USP28 or SREBP2, with several of the overlapping genes also found among the significantly regulated proteins (Fig. 1g). Together, these data indicate that USP28 alters the expression of SREBP2 target genes, particularly those mapping to the MVP.

We next applied stable isotope labelling using U-^13^C-Glucose to follow MVP metabolite flux in A431 cells. Surprisingly, the cholesterol pool remained largely unlabelled, suggesting that these cells obtain cholesterol mostly through uptake rather than de novo synthesis (Fig. 1h). In contrast, a large proportion of ubiquinone (CoQ10), a metabolite containing ten isoprenyl subunits in its side-chain, showed a high degree of labelling. Depletion of USP28 significantly reduced the proportion of labelled CoQ10, indicating downregulation of the MVP, while cholesterol labelling was only mildly affected (Fig. 1i). MVP regulation by USP28 was also confirmed in U2OS osteosarcoma cells, where knockout of USP28 reduced expression of HMGCS1 and resulted in a small but significant decrease in cholesterol levels (Fig. S1h, i).

### SREBP2 associates and colocalises with USP28

**Fig. 2:**
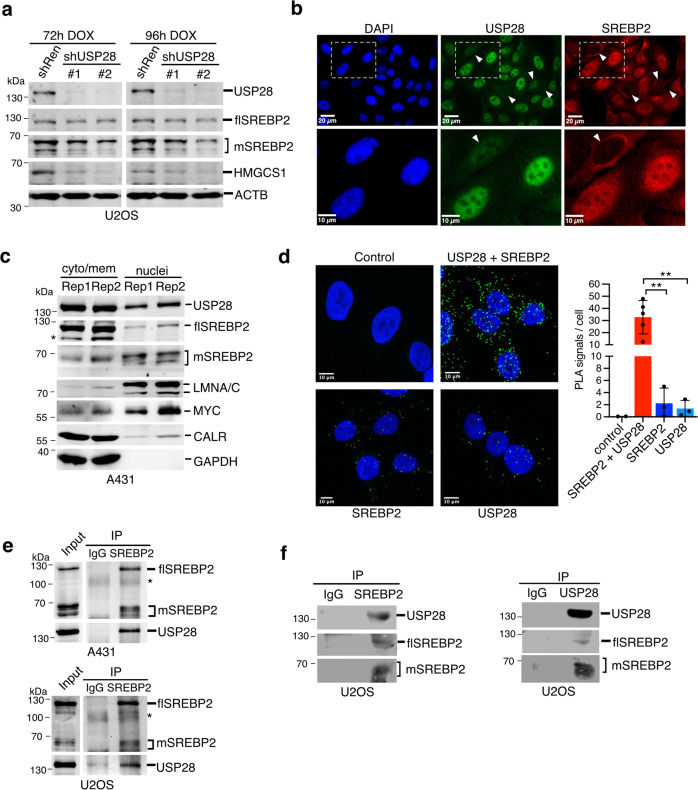
USP28 regulates SREBP2 via close interaction. **a** U2OS cells expressing inducible shRNA sequences targeting USP28 (shUSP28#1 and shUSP28#2) or non-targeting control (shRen) were treated with 1 µg/ml of doxycycline (DOX) for 72 or 96 h. Protein lysates were analysed for expression of USP28, full length (flSREBP2) and mature SREBP2 (mSREBP2) and HMGCS1 by immunoblotting. Actin is shown as loading control. **b** U2OS cells were stained for USP28 and SREBP2 by immunofluorescence. DAPI was used to mark nuclei. Cells with low nuclear expression of USP28 and SREBP2 are marked with arrowheads. **c** A431 cells were subjected to cell fractionation into cytoplasm and nuclei. Fractions were analysed for expression of USP28 as well as full length (flSREBP2) and mature SREBP2 (mSREBP2). Calreticulin served as marker for ER-membranes, c-Myc and Lamin A/C as markers for nuclei and GAPDH as marker for cytoplasm. Unspecific bands for flSREBP2 are marked by asterisks. Two independent replicate fractionations are shown. **d** U2OS cells were analysed by proximity ligation assay using antibodies specific for USP28 and SREBP2 either alone or in combination. Number of PLA signals per cell were analysed in >42 cells from at least three biologically independent samples. Data are presented as mean ± SD (^*^p < 0.05, ^**^p < 0.01, one-way ANOVA with post-hoc Dunnett’s test). **e** A431 and U2OS cells were subjected to cross-linking with DSP. Cells were fractionated and lysates were subjected to immunoprecipitation using antibodies detecting SREBP2 or matched immunoglobulin controls (IgG). Input and precipitates were analysed for presence of full length and mature SREBP2 as well as USP28. IgG bands are marked by asterisks. **f** Lysates of U2OS cells were subjected to immunoprecipitation using antibodies detecting SREBP2 (left) or USP28 (right). Precipitates were analysed for presence of full-length and mature SREBP2 as well as USP28.

As USP28 silencing resulted in downregulation of SREBP2 target genes without affecting expression of *SREBF2* mRNA (Fig. S1d), we next investigated whether USP28 regulates SREBP2 protein level. Immunoblot analysis of USP28-silenced U2OS cells (Fig. 2a) revealed a marked reduction in expression of HMGCS1 as well as a loss of the mature form of SREBP2, which forms multiple bands of approximately 65–70 kDa due to post-translational modifications [32]. In contrast, the 125 kDa full-length SREBP2 protein was not affected. Immunofluorescence staining showed mostly nuclear localisation of USP28, while SREBP2 is found both in the nucleus and in a perinuclear region (Fig. 2b), likely representing the ER-membrane. Moreover, individual cells displaying low USP28 staining (marked by arrowheads) were mostly devoid of nuclear SREBP2 (Fig. 2b), with nuclear staining intensities of USP28 and SREBP2 showing positive correlation (Fig. S2a). Cell fractionation in A431 cells revealed that USP28 and full length SREBP2 were found in the cytoplasm and cytoplasmic membranes (Fig. 2c), as indicated by the presence of the ER-membrane protein calreticulin (CALR) and cytoplasmic GAPDH. USP28 also colocalised with mature SREBP2 in the nuclear fraction, identified by the presence of MYC and lamin A/C (Fig. 2c).

We next employed proximity ligation assays to investigate association of the two proteins in situ. Indeed, a strong enhancement of nuclear and cytoplasmic PLA signals was observed when the probes for USP28 and SREBP2 were combined (Fig. 2d). USP28 was also detected in SREBP2 immunoprecipitates from lysates of A431 and U2OS cells (Fig. 2e) and both proteins could be coprecipitated from U2OS cells (Fig. 2f). Combined, these results indicate that USP28 and SREBP2 co-localise and interact, suggesting that USP28 could function as a deubiquitinating enzyme for SREBP2. However, it is possible that the interaction between SREBP2 and USP28 is indirect and involves additional, unknown proteins.

### USP28 deubiquitinates and stabilises SREBP2

**Fig. 3:**
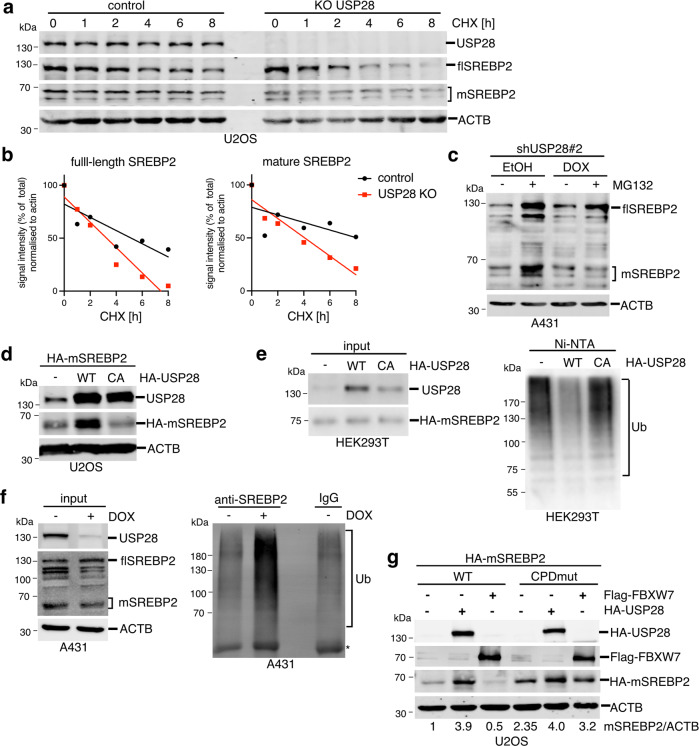
USP28 controls the stability of mature SREBP2. **a** U2OS cells were transfected with gRNAs targeting USP28 (KO USP28) and parental controls were treated with 50 µg/ml cycloheximide for the indicated times. Levels of USP28 as well as full length (flSREBP2) and mature SREBP2 (mSREBP2) were determined by immunoblotting. Actin is shown as loading control. **b** Quantification of full-length and mature SREBP2 signals relative to actin from (**a**). **c** A431 cells expressing inducible shRNA sequences targeting USP28 (shUSP28#2) were treated with 0.5 µg/ml of doxycycline (DOX) or solvent (ethanol, EtOH) for 72 h. During the last 6 h, 20 µM MG-132 was added. Cells were lysed and analysed for expression of full-length (flSREBP2) and mature SREBP2 (mSREBP2). Actin is shown as loading control. **d** U2OS cells were transfected with expression vectors coding for HA-tagged mature SREBP2 (aa 1-484, HA-mSREBP2) together with HA-tagged wild type (WT) or catalytically inactive (CA) USP28. Levels of USP28 and mature SREBP2 were determined by immunoblotting. Actin is shown as loading control. **e** HEK293 cells were transfected with expression vectors coding for HA-tagged USP28 or HA-tagged mSREBP2 together with His6-Ubiquitin (K48-only). Ubiquitinated proteins were purified using Ni-NTA and analysed by immunoblotting (Ub). Presence of USP28 and SREBP2 was confirmed in the input. **f** A431 cells expressing inducible shRNA sequences targeting USP28 (shUSP28#2) were treated with 1 µg/mL doxycycline (DOX) or solvent (ethanol) for 96 h with 20 µM MG132 being added during the last 6 h. Cells were lysed and subjected to immunoprecipitation using antibodies detecting SREBP2. Precipitates were analysed for the presence of ubiquitinated proteins (Ub). Efficient silencing of USP28 and presence of full length and mature SREBP2 was confirmed in the input. Actin is shown as a loading control. IgG bands are marked by asterisks. **g** U2OS cells were transfected with either wild type or CPD mutant (Ser 432 and 436 to Ala) HA-tagged mature SREBP2 together with HA-tagged USP28 or Flag-FBXW7. Levels of USP28, FBXW7 and mature SREBP2 were determined by immunoblotting. Actin is shown as loading control.

We next determined whether USP28 regulates SREBP2 stability. First, we treated USP28 wild-type or KO U2OS cells with cycloheximide (CHX) to block translation in a time-course experiment. Deletion of USP28 accelerated the loss of both full-length and mature SREBP2 compared to wild-type cells (Fig. 3a, b). Similar results were also obtained after USP28 silencing (Fig. S3a, b). Furthermore, silencing of USP28 in A431 prevented the stabilisation of mature SREBP2 following proteasome inhibition by MG-132 (Fig. 3c).

To address the mode of regulation of SREBP2 by USP28, we used U2OS cells to co-express mature SREBP2 together with either wild-type USP28 (WT) or a mutant variant, in which the catalytic cysteine (Cys 171) within the USP domain was replaced by alanine (CA). This showed that wild-type USP28 increases the levels of mature SREBP2, which was not observed with the CA mutant (Fig. 3d). USP28 WT but not CA mutant also enhanced levels of c-Myc and mature SREBP1a (Fig. S3c, d), indicating a similar mode of regulation. Furthermore, USP28 WT but not CA mutant decreased levels of mature SREBP2 in a pull-down assay using 6His-tagged K48-only ubiquitin (Fig. 3e), while silencing of USP28 increased the amount of SREBP2 ubiquitination detected by immunoprecipitation in A431 cells (Fig. 3f).

FBXW7 has been implicated in the ubiquitination and destabilisation of both SREBP1 and SREBP2 [21] and several established targets of USP28 are also substrates for FBXW7 [33]. Interestingly, deletion of the CPD motif in SREBP2, which abolished the destabilisation of SREBP2 by FBXW7, only had a partial effect on the ability of USP28 to stabilise SREBP2 (compare 3.9- to 1.7-fold increase in mSREBP2 protein levels, Fig. 3g). This was in contrast to similar experiments performed with SREBP1, where mutation of the CPD completely blocked regulation by both FBXW7 and USP28 (Fig. S3e). This suggests that USP28 might also target ubiquitin residues in SREBP2 introduced by other ubiquitin ligases, potentially explaining the strong stabilising effect on this transcription factor.

### Depletion of USP28 renders cancer cells highly sensitive to MVP inhibition

**Fig. 4:**
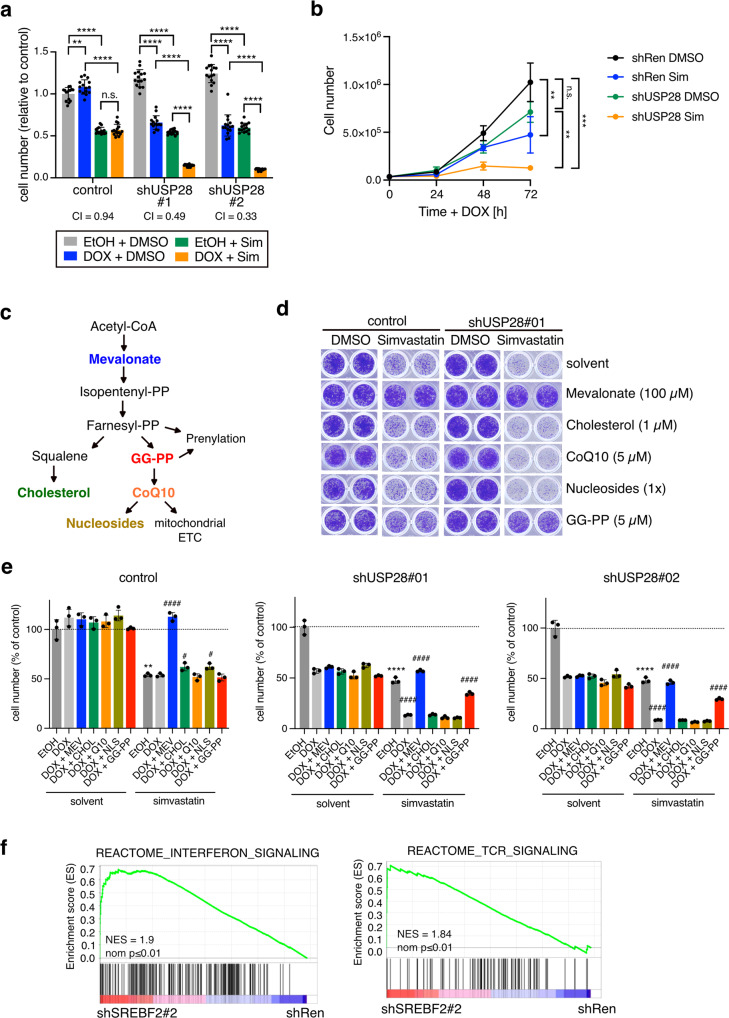
Inhibition of USP28 and MVP synergise in reducing cell viability. **a** A431 cells expressing inducible shRNAs targeting USP28 (shUSP28#1 or shUSP28#2) or control cells were treated with 1 µg/ml doxycycline (Dox) or solvent (ethanol, EtOH) for 72 h together with 10 µM simvastatin (Sim) or solvent (DMSO). Cell viability was determined by crystal violet staining. Data are presented as mean± SD of three independent experiments (n.s. non-significant, ^****^p < 0.00001, one-way ANOVA with post-hoc Tukey test). **b** Growth curves of shUSP28#1 or control (shRen) treated with 1 µg/ml doxycycline (Dox) or solvent (ethanol, EtOH) together with 10 µM simvastatin (Sim) or solvent (DMSO). Data are presented as mean± SD of three independent experiments (n.s. non-significant, ^*^p < 0.05, ****p < 0.00001, one-way ANOVA with post-hoc Tukey test). **c** Schematic of the different metabolic outputs of the MVP. GG-PP = geranyl-geranyl-pyrophosphate, CoQ10 = ubiquinone. **d** Cells as in (**a**) were treated with 1 µg/ml doxycycline or solvent (ethanol, EtOH) for 72 h together with 10 µM simvastatin (Sim) or solvent (DMSO) plus the indicated compounds: 100 µM mevalonate, 1 µM cell-permeable cholesterol, 5 µM ubiquinone (CoQ10), nucleosides (30 µM C, G, A, U each; 10 µM T) or 5 µM GG-PP. Cell viability was determined by crystal violet staining. Representative images of three independent replicates are shown. **e** Quantitation of data shown in (**d**). Data are presented as mean± SD of three independent replicates (n.s. non-significant, ^**^p < 0.01, ^****^p < 0.0001, unpaired two-tailed Student’s *t*-test between EtOH vs EtOH plus simvastatin; ^#^*p* < 0.05, ^####^*p* < 0.0001, one-way ANOVA with post-hoc Dunnett’s test compared to DOX plus simvastatin). **f** GSEA enrichment plots for gene sets mapping to interferon and TCR signalling comparing cells depleted for *SREBF2* (SREBF2#2) and control (shRen).

Having identified USP28 as a regulator of SREBP2, we next investigated whether targeting the USP28/SREBP2 axis would affect viability of squamous cancer cells. Consistent with previous findings [10], we observed that silencing of USP28 reduced colony formation in A431 cells (Fig. S4a, b). Interestingly, USP28 silencing rendered A431 cells highly sensitive to MVP inhibition by the HMGCR-inhibitor simvastatin (Fig. 4a, b), suggesting cooperation between the two pathways. While USP28 silencing or simvastatin treatment alone resulted in an approximately 50% reduction, combining these two treatments resulted in an 80–90% reduction in cell number (combination index 0.49 and 0.33 for shUSP28#1 and shUSP28#2, respectively).

The MVP has multiple metabolic outputs that participate in numerous different cellular processes, including protein prenylation and the mitochondrial electron transfer chain (ETC) (Fig. 4c) [20]. We have shown previously that the MVP supplies ubiquinone (CoQ10) to support enhanced pyrimidine nucleotide biosynthesis in TP53 deficient colon cancer cells [34]. To determine which MVP output is crucial for cell survival, we treated *USP28*-silenced A431 cells with simvastatin in combination with either mevalonate, cell-permeable cholesterol, CoQ10, nucleosides or geranyl-geranyl-pyrophosphate (GG-PP), the substrate for the prenylation of small GTPases [35, 36]. Mevalonate restored cell viability of simvastatin treated cells, confirming the specificity of the drug (Fig. 4d, e). In contrast to the other treatments, only GG-PP restored cell viability at least partially, indicating that the provision of substrates for protein prenylation is an essential output of the MVP in these cells.

In order to explore potential mechanisms downstream of SREBP2 in SCC cells, we further analysed the RNAseq data from A431 cells after *SREBF2* silencing. Interestingly, *SREBF2* depletion caused a marked induction of gene expression signatures connected to interferon and T cell receptor signalling (Fig. 4f). Among the strongest regulated genes within these gene sets were those coding for major histocompatibility complex class I and II (MHC-I, MHC-II) proteins (Fig. S4c), suggesting that depletion of *SREBF2* could trigger enhanced antigen presentation. This could be reminiscent of a mechanism recently described for pancreatic cancer where deletion of oncogenic *Kras* blocked immune evasion via the induction of MHC gene expression [37].

### USP28 and SREBP2 are overexpressed in SCC

**Fig. 5:**
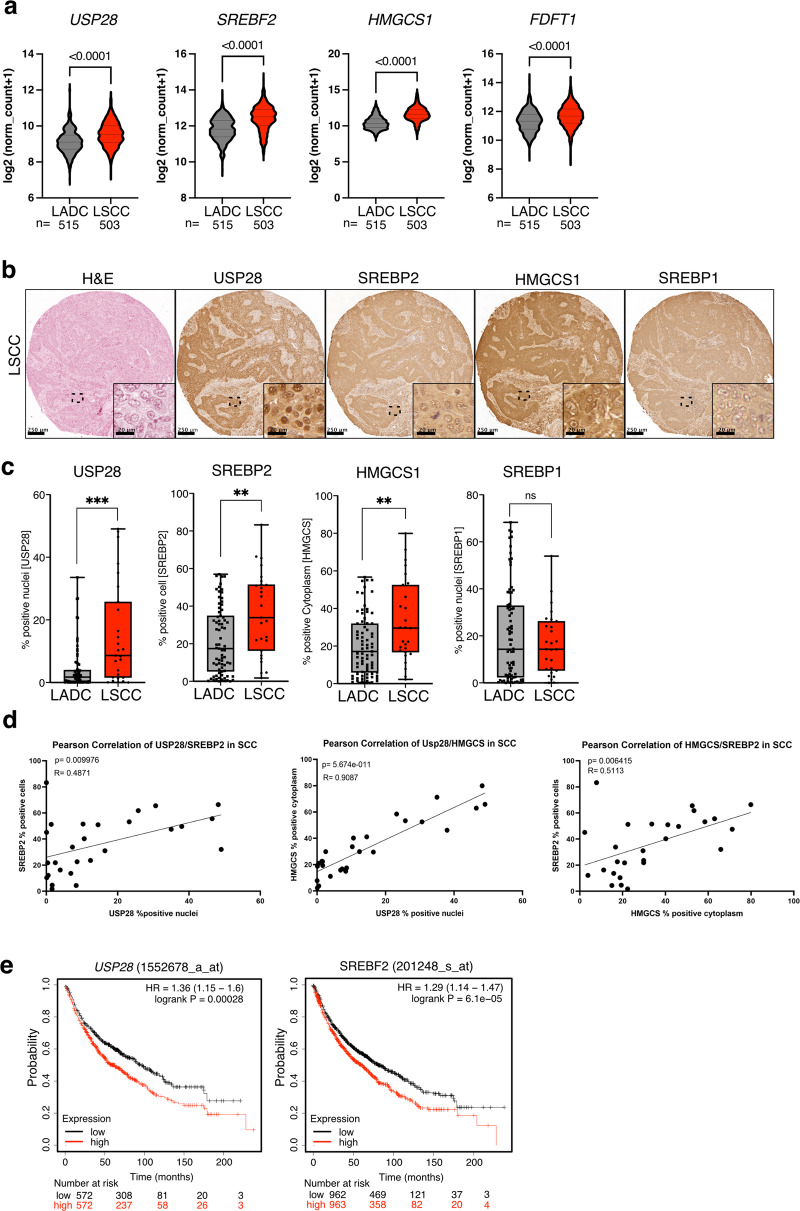
SREBP2 is upregulated in squamous cell carcinoma. **a** Violin plots showing expression of *USP28*, *SREBF2, HMGCS1* and *FDFT1* in human Lung adenocarcinoma (ADC) and lung squamous carcinoma (SCC). Data are from TCGA (Firehose legacy). Significance was calculated using Mann–Whitney test. ^****^p < 0.0001. **b** Tissue sections from a human NSCLC TMA were stained for USP28, SREBP2 HMGCS1 and SREBP1 by immunohistochemistry. Haematoxilin and eosin (H&E) staining is also shown. Representative images for SCC are shown. **c** Boxplots showing quantification of USP28, SREBP2, HMGCS and SREBP1 staining in SCC and ADC tumours. Percent positive cells are shown. (SCC: *n* = 33; ADC: *n* = 75; ^***^p < 0.001, Mann-Whitney test). **d** Pearson correlation between expression of USP28, SREBP2 and HMGCS in SCC tumours (R = Pearson correlation coefficient, p = two tailed t-test). **e** Kaplan–Meier plots of overall survival of lung cancer patients (*N* = 1925) dichotomised into ‘high’ and ‘low’ based on median *USP28* or *SREBF2* expression score. Survival differences were calculated with the log-rank test.

USP28 has been identified as a major oncogenic regulator in squamous cell carcinoma [33] and its genetic deletion or inhibition was shown to block tumour growth in mouse models of LSCC [16, 17]. Analysis of TCGA data revealed that a set of cholesterol biosynthesis genes showed positive correlation with *USP28* expression in cervical squamous cell carcinoma and endocervical adenocarcinoma (CESC), head and neck squamous cell carcinoma (HNSC) as well as in LSCC, but not in normal lung tissue (Fig. S5a). Furthermore, expression of *USP28*, *SREBF2*, *HMGCS1* and *FDFT1* was significantly higher in LSCC compared to LADC (Fig. 5a). It should be noted that SREBP2 can also induce transcription from the *SREBF2* gene via a sterol response element (SRE) located in its promoter [38]. Thus, stabilisation of mature SREBP2 by USP28 can also enhance *SREBF2* mRNA expression.

We next analysed a tissue microarray comprising over 100 tissue samples of human non-small lung cancer by immunohistochemistry combined with quantitative image analysis (QuPath). This revealed that USP28, SREBP2 and HMGCS1, but not SREBP1, were significantly higher expressed in LSCC tumours (*n* = 33) compared to LADC tumours (*n* = 75) (Figs. 5b, c, S5b). Furthermore, nuclear staining for USP28 showed positive correlation with SREBP2 and HMGCS2 expression in LSCC but not in LADC (Figs. 5d and S5c). Finally, Kaplan–Meier analysis of human lung cancer patient data revealed that both *USP28* and *SREBF2* were indicative of poor survival in a mixed cohort (Fig. 5e). Individual analysis showed that LUAD patients with high SREBF2 expression show poor overall survival, which is similar to that of LSCC patients (Fig. S5d). Together, these data indicate that both USP28 and SREBP2 are upregulated in human squamous cell carcinoma, particularly the LSCC subtype.

### Deletion of SREBF2 reduces tumour formation in a LSCC mouse model

To study the role of SREBP2 in LSCC, we employed a CRISPR/Cas9 mouse model in which lung tumours are induced by intratracheal delivery of adeno-associated virus (AAV) particles into *Rosa26:Cas9* transgenic mice (Fig. S6a). This strategy was used to introduce the oncogenic mutation G12D into the *Kras* locus via a repair template in combination with inactivation of *Tp53* either alone (KP) or together with a *Stk11/Lkb1* deletion (KPL). We have previously shown that KPL mice develop LSCC at high frequency, while only a minority of lesions present with a LADC phenotype [16]. In this model, deletion of *Usp28* prevented LSCC formation, resulting in a reduced tumour load and enhanced survival [16].

**Fig. 6:**
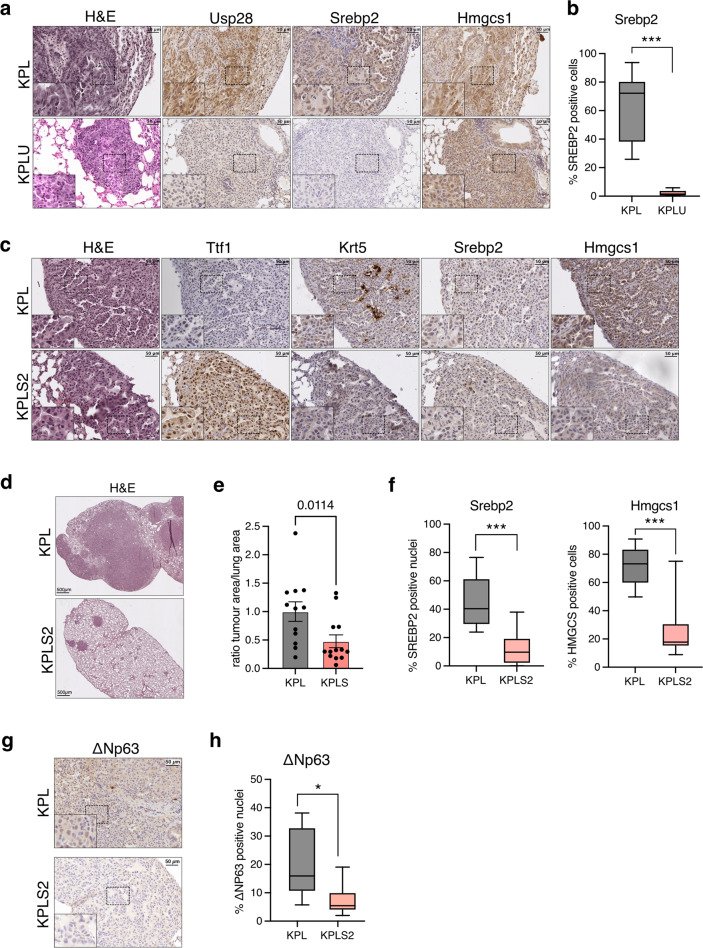
Deletion of *Srebf2* attenuates tumour formation in a mouse model of lung squamous cell carcinoma. **a** Tissue sections from KPL and KPLU lung tumours were stained for Usp28, Srebp2 and Hmgcs1 by immunohistochemistry. Haematoxylin and eosin (H&E) staining is also shown. **b** Boxplot showing quantification of Srebp2 staining in KPL and KPLU tumours. Percent positive cells are shown. (KPL: *n* = 9; KPLU: *n* = 7; ^***^p < 0.001, Mann–Whitney test). **c** Tissue sections from KPL and KPLS2 tumours were stained for the adenocarcinoma marker thyroid transcription factor 1/NK2-homeobox 1 (Ttf-1/Nkx2-1), the squamous marker keratin 5 (Krt5), Srebp2 and Hmgcs1 by immunohistochemistry. H&E staining is also shown. **d** H&E staining of representative lung tissue sections from KPL and KPLS2 mice. **e** Ratio of tumour area relative to total lung area in KPL and KPLS2 mice. Data are displayed as mean ± SD (KPL: *n* = 11; KPLS2: *n* = 13; Mann-Whitney test). **f** Boxplot showing quantification of Srebp2 and Hmgcs1 staining in KPL and KPLS2 tumours. Percent positive nuclei or positive cells are shown. (KPL: *n* = 7; KPLS2: *n* = 9; ^***^p < 0.001, Mann-Whitney test). **g** Tissue sections from KPL and KPLS2 tumours were stained for ΔNp63. **h** Boxplot showing quantification of ΔNp63 staining in KPL and KPLS2 tumours. Percent positive nuclei are shown. (KPL: *n* = 6; KPLS2: *n* = 6; ^***^p < 0.05, Mann-Whitney test).

We first investigated lung tissue sections from KP and KPL mice by immunohistochemistry staining. This revealed that in both KP and KPL mice, tumour lesions showed upregulation of the Srebp2 target Hmgcs1 (Fig. S6b–e). Tumours from KPL mice also displayed a high percentage of nuclei that were positive for Usp28 (Fig. S6d, e). We next compared tumours from the SCC and ADC subtypes, as determined by keratin 5 (Krt5), thyroid transcription factor 1 (Ttf-1/Nkx2-1) and Usp28 staining, in KPL mice. Interestingly, while both tumour subtypes showed increased Hmgcs1 levels compared to normal tissue, Hmgcs1 expression was significantly higher in SCC compared to ADC (Fig. S6f, g). We also analysed lung tumour tissues after CRISPR/Cas9 deletion of Usp28 in KPL tumours (KPLU). These tumours showed a marked drop in the percentage of nuclei that are positive for SREBP2 (Fig. 6a, b). Furthermore, expression of HMGCS1 and FDFT1 was also reduced in lysates from KPLU compared to KPL tumours (Fig. S6h), providing additional evidence that Usp28 regulates Srebp2 in lung cancer.

We, therefore, used the same AAV-based CRISPR/Cas9 strategy to target *Srebf1* or *Srebf2* in KPL tumours (KPLS1 and KPLS2). The efficiency of the gRNAs used to delete *Srebf1* or *Srebf2* was first established using T7E1 analysis and immunoblotting (Fig. S5e, f). While deletion of *Srebf1* did not alter tumour load (Fig. S6i), deletion of *Srebf2* resulted in a significant reduction in tumour burden in age matched KPLS2 compared to KPL mice (Fig. 6d, e). We also observed a significant reduction in the percentage of Srebp2 positive nuclei and Hmgcs1 positive cells (Fig. 6f), confirming efficient target deletion. Consistent with our observation that SREBP2 regulates expression of TP63 targets in human SCC cells (see Fig. S1e), we also observed a reduction in ΔNp63 positive nuclei in KPLS2 tumours (Fig. 6g, h). Together, these results strongly implicate SREBP2 in lung tumour growth and suggest that SREBP2 contributes to SCC transcriptional programmes via regulating ΔNP63.

### Statins synergise with a dual USP28/25 inhibitor to reduce viability in SCC cells

**Fig. 7:**
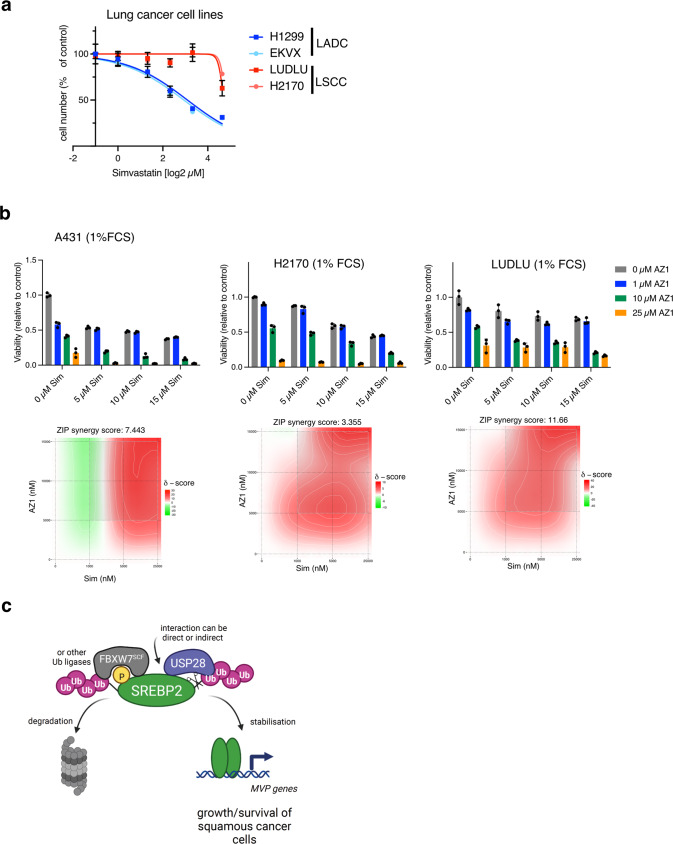
Simvastatin synergises with a dual USP28/25 inhibitor to reduce viability of SCC cells. **a** Human ADC (H1299 and EKXV) and SCC (LUDLU and H2170) cells were treated with different concentrations of simvastatin for 72 h. Cell viability was determined using crystal violet staining. **b** A431, H2170 and LUDLU cells were treated with indicated concentrations of simvastatin (Sim) or AZ1 in medium containing 1% FCS for 72 h. Drug synergy was calculated using ZIP model. **c** Working model for the regulation of SREBP2 by USP28. Created with BioRender.com.

In order to investigate the translational significance of targeting the USP28/SREBP2 axis in squamous cancer, we first investigated whether LADC and LSCC cell lines respond different to inhibition of the MVP by statins. While LADC cells (H1299 and EKVX) were highly sensitive to simvastatin treatment, two LSCC cell lines (LUDLU and H21170) showed a remarkable resistance to this drug (Fig. 7a). We, therefore, treated A431 and the two LSCC cell lines with a combination of simvastatin and the dual USP28/25 inhibitor AZ1 [39]. We also restricted exogenous lipid availability by culturing cells in 1% FCS. Interestingly, drug synergy between simvastatin and AZ1 was only observed when cells were exposed to lipid deplete conditions (Figs. 7b and S7a). These results indicate that combined targeting of USP28 and MVP could be efficient in eliminating SCC cells.

## Discussion

SREBP transcription factors integrate multiple cellular signals to drive the expression of genes controlling fatty acid and cholesterol biosynthesis [19, 20]. Here we have investigated the regulation of SREBP2 by the deubiquitinating enzyme USP28. We found that depletion of USP28 reduces the expression of multiple MVP proteins, most prominently enzymes that catalyse the sequential condensation of acetoacetyl-CoA to form isoprenoid chains that are used for protein prenylation and for the synthesis important cellular metabolites, including CoQ10, dolichol and cholesterol. Using ^13^C-glucose tracing, we found that A431 cells display very low levels of de novo cholesterol biosynthesis but divert glucose-derived carbons into the synthesis of isoprenoids. We have recently shown that squalene epoxidase (SQLE) is upregulated in aggressive prostate cancer through a micro-RNA dependent mechanism to drive de novo cholesterol biosynthesis [40]. Our current findings suggest that different cancer entities distinctly modulate critical nodes of the MVP to fulfil specific metabolic demands.

Our results also demonstrate that USP28 regulates the MVP by increasing the stability of SREBP2, the transcription factor responsible for the expression of most MVP enzymes. While both proteins localise to and interact in the nucleus, we did observe some level of interaction of SREBP2 and USP28 also in the cytoplasm. So far, most studies have described USP28 as a primarily nuclear protein. One potential explanation for cytoplasmic localisation could be alternative splicing, leading to broader subcellular distribution of USP28. Indeed, alternatively spliced products of USP28 have been described [41]. Nevertheless, USP28-dependent SREBP2 stabilisation most likely targets the mature form, as it was detected in our experiments when only the isolated N-terminal part of the protein was expressed. Interestingly, mutation of the CPD, which mediates GSK3β-dependent phosphorylation and subsequent recognition by the SCF/FBXW7 ubiquitin ligase, did not fully abolish the regulation of SREBP2 by USP28. This suggests that mature SREBP2 can also be a substrate for other ubiquitinating enzymes. An early study described degradation of nuclear SREBPs by the ubiquitin-proteasome system without identifying the ubiquitin ligases involved [42]. The only other ubiquitin ligases, in addition to FBXW7, described to regulate SREBP2 so far are TRC8 (also known as ring finger protein 139, RNF139) and ITCH (Itchy E3 Ubiquitin Protein Ligase). TRC8 ubiquitinates the full-length protein to prevent SREBP2 processing [43, 44], while ITCH potentially targets nuclear SREBP2 [45]. While further investigations are needed to identify additional ubiquitin ligases for SREBP2, the possibility that USP28 might also target ubiquitin chains assembled by other ubiquitin ligases on SREBP2 could explain the strong effect of USP28 on SREBP2 stability. Our working model for the regulation of SREBP2 by USP28 is displayed in Fig. 7c. Notably, it is unclear whether the interaction between SREBP2 and USP28 is direct or whether it involves additional proteins.

USP28 is highly upregulated in LSCC, where it stabilises the transcription factor ΔNP63 that is essential for the maintenance of the squamous lineage [16]. We observed that USP28 depletion reduced the viability of A431 SCC cells and rendered them highly sensitive to MVP inhibition by simvastatin and that this effect could be partially rescued by the protein prenylation substrate GG-PP. It has been previously shown that inhibition of SREBP by the retinoblastoma protein leads to reduced prenylation of NRas [46]. Furthermore, production of GG-PP by the MVP has been linked to Rho activation to drive the disruption of tissue architecture by mutant p53 [47]. As activating mutations in Ras GTPases are frequently found in squamous cancers [48, 49], it is possible that the MVP provides essential substrates for protein prenylation to support Ras and Rho signalling in SCC cells.

We also found positive correlation in expression of *USP28* and *SREBF2* in several SCC entities, including LSCC. Moreover, USP28, SREBF2 and HMGCS1 were significantly upregulated in LSCC compared to LADC in tissue from a cohort of human lung cancer patients. Furthermore, expression of USP28 and SREBP2 or HMGCS1 showed positive correlation in LSCC tissue, providing further evidence for their coregulation. Furthermore, Krt5^high^/Ttf-1^low^ lesions from a CRISPR-based lung cancer model driven by oncogenic activation of *Kras* together with deletion of *Tp53* and *Lkb1* (KPL) representing squamous tumours displayed increased levels of HMGCS1 expression compared to tumours mapping to the ADC subtype. Genetic deletion of *Usp28*, which has previously been shown to lower ΔNp63 and reduce tumour formation in mouse SCC, from these tumours (KPLU) also dramatically decreased levels of nuclear Srebp2. Critically, CRISPR/Cas9-mediated deletion of *Srebf2* from KPL tumours (KPLS2) resulted in a marked reduction in tumour load and reduced expression of the SCC marker ΔNp63. Finally, we observed that treatment with a dual USP28/25 inhibitor restored sensitivity of SCC cells towards MVP inhibition using statins, with drug synergy being observed when cells were exposed to lipid-deplete conditions.

Together, our data provide strong evidence that SREBP2 is essential for cancer cell survival in SCC. A recent metabolomics study found increased levels of cholesterol in tissue samples from oral squamous cell carcinoma [50]. SREBP2 was previously found to be upregulated in oesophageal squamous cancer (ESCC) where it supports cancer cell viability, migration and invasion [51]. Furthermore, activation of cholesterol synthesis by LPCAT was shown to promote ESCC progression [52]. It should be noted that a recent study reported that SREBP1 cooperates with TP63 and KLF5 to drive fatty acid metabolism and other SCC-specific gene expression signatures [53]. We observe here that SCC cells respond to SREBP2 silencing with a prominent downregulation of a TP63 target gene signature. Furthermore, we found that deletion of Srebf2 from LSCC tumours was associated with reduced nuclear expression of ΔNP63. These results suggest that SREBP2 controls SCC-related transcriptional programmes. Interestingly, SREBP2 silencing was also associated with a strong induction of pro-inflammatory signatures, including IFN and T cell receptor signalling. It has been shown that limiting flux through the cholesterol biosynthesis pathway leads to the activation of type I IFN response by conformational activation of STING in the ER-membrane of macrophages [54]. It is also possible that impaired prenylation of RAS or RHO proteins leads to the activation of IFN signalling, for example by triggering ER-stress as reported previously [55]. It will also be interesting to investigate whether combinatorial treatment using the more selective USP28 inhibitors that are currently under pre-clinical evaluation [17] together with some of the established therapeutics targeting MVP activity [56] could exacerbate the induction of inflammation and stress signalling, resulting in improved anti-tumour effects.

While further experiments are required to reveal the full complexity of cancer-relevant pathways controlled by USP28 and SREBP2, the results provided by our study add an additional layer to the regulation of SREBP2 activity and open novel translational avenues for the treatment of squamous tumours.

## Supplementary information

Supplemental material

Uncropped WB images

Reporting summary

## Data Availability

All data and material will be made available upon request. Original images of all western blots are provided as supplementary material.
