# Supplementary material for: USP28 controls SREBP2 and the mevalonate pathway to drive tumour growth in squamous cancer
Source: Cell Death Differ. 2023 May 18;30(7):1710–25. doi: 10.1038/s41418-023-01173-6 (PMC10307777; doi:10.1038/s41418-023-01173-6)
Supplement: Supplementary file 1 — Supplemental material [file 41418_2023_1173_MOESM1_ESM.pdf]

Maier et al. Supplementary Figures and Legends

Supplementary Figure S1: Related to Figure 1

a) Differential expression of MVP proteins as determined by proteomics analysis. Table shows log2 fold change (log2FC), p-value and q-value (unpaired, parametric Student t-test with Benjamini/Hochberg FDR) of signal intensities in shUSP28 expressing cells compared to non-targeting controls.

b) A431 cells expressing inducible shRNA sequences targeting USP28 (shUSP28#1 and shUSP28#2) or non-targeting control (shRen) were treated with 0.5µg/ml of doxycycline (DOX) for 96 hours. Gene expression of *USP28* and *HMGCS2* were analysed by qPCR.

c) A431 cells expressing inducible shRNA sequences targeting SREBF2 (shSREBF2#1 and shSREBF2#2) or non-targeting control (shRen) were treated with 1µg/ml of doxycycline (DOX) for 96 hours. During the last 24 hours, medium was replaced to 10% FCS or 1% FCS. Expression of SREBP2 and HMGCS1 was analysed by immunoblotting. Actin is shown al loading control.

d) Expression of *SREBF2* and *HMGCS1* mRNA in A431 cells depleted of SREBF2 or USP28.

e) A431 cells expressing an inducible shRNA sequence targeting SREBF2 (shSREBF2#1) or non-targeting control (shRen) were treated with 1µg/ml of doxycycline (DOX) for 120 hours. Changes in gene expression were analysed and enrichment plots for gene sets mapping to cholesterol biosynthesis and direct ΔNP63 targets are shown.

f) Heatmaps showing expression of genes linked to cholesterol synthesis in A4341 cells depleted of USP28 or SREBF2.

g) Heatmaps showing expression of direct ΔNP63 targets genes in A4341 cells depleted of USP28 or SREBF2.

h) U2OS cells were transiently transfected with gRNAs targeting USP28 (KO USP28, clone 2 and clone 6) together with Cas9 and compared to control cells. Expression of USP28 and HMGCS1 was analysed by immunoblotting. Vinculin is shown as loading control.

i) Extracts from parental or USP28 KO (c2) cells were analysed for levels of cholesterol using LC-MS. Data are displayed as mean ± SD from 3 independent replicates. Significance was calculated using unpaired two-tailed Student's t-test.

Supplementary Figure S2: Related to Figure 2

a) Quantitation of immunofluorescence data shown in Figure 2b.

Supplementary Figure S3: Related to Figure 3

a) U2OS cells expressing inducible shRNAs targeting USP28 were treated with 1 µg/ml doxycycline or ethanol (EtOH) for 72 hours and subsequently with 50 µg/ml cycloheximide for

the indicated times. Levels of USP28 as well as full length (flSREBP2) and mature SREBP2 (mSREBP2) were determined by immunoblotting. Vinculin is shown as loading control.

b) Quantification of total SREBP2 signal relative to vinculin from (a).

c) U2OS cells were transfected with expression vectors coding for HA-tagged c-Myc (HA-MYC) together with HA-tagged wild type (WT) or catalytically inactive (CA) USP28. Levels of USP28 and c-Myc were determined by immunoblotting. Actin is shown as loading control.

d) U2OS cells were transfected with expression vectors coding for myc-tagged mature SREBP1a (amino acids 1-490) (myc-SREBP1a) together with HA-tagged wild type (WT) or catalytically inactive (CA) USP28. Levels of USP28 and SREBP1a were determined by immunoblotting. Actin is shown as loading control.

e) U2OS cells were transfected with either wild type or CPD mutant (Ser 432 and 436 to Ala) HA-tagged mature SREBP1 together with HA-tagged USP28 or Flag-FBXW7. Levels of USP28 and mature SREBP1 were determined by immunoblotting. Actin is shown as loading control.

Supplementary Figure S4: Related to Figure 4

a) A431 cells expressing inducible shRNAs targeting *USP28* (shUSP28#1 and shUSP28#2) or non-targeting controls (shRenilla) were seeded at low density and treated with 1 µg/ml doxycycline (DOX) for 6 days. Representative images are shown.

b) Quantitation of data shown in a. (**p<0.01, one-way ANOVA with post-hoc Dunnett's test).

c) Heat maps showing the regulation of genes mapping to interferon and TCR signalling in A431 cells following *SREBF2* silencing.

Supplementary Figure S5: Related to Figure 5

a) Correlation between expression of *USP28* and 14 genes mapping to the cholesterol biosynthesis pathway (CholBiosyn) in normal lung tissue, cervical squamous cell carcinoma and endocervical adenocarcinoma (CESC), head and neck squamous cell carcinoma (HNSC) and lung squamous cell carcinoma (LSCC). TPM = transcripts per million. R=Spearman correlation, *pValue*=0.53. Data was generated using the online tool http://gepia2.cancer-pku.cn/#index.

b) Tissue sections from a human NSCLC TMA were stained for USP28, SREBP2 HMGCS1 and SREBP1 by immunohistochemistry. Haematoxilin and eosin (H&E) staining is also shown. Representative images for ADC are shown.

c) Pearson correlation between expression of USP28, SREBP2 and HMGCS in ADC tumours (R = Pearson correlation coefficient, p = two tailed t-test).

d) Kaplan-Meier plots of overall survival of LADC (*N* = 791) and LSCC (N = 524) patients dichotomised into 'high' and 'low' based on median *SREBF2* expression score. Survival differences were calculated with the log-rank test.

e) Murine hepatocellular carcinoma (mHCC) cells were infected with lentiviruses encoding guide RNAs for *SREBF1* and *SREBF2*, respectively, together with Cas9. Genomic DNA was extracted and cleaved with T7-endonuclease. Cleavage products indicative of efficient gene disruption was analysed on agarose gel.

f) Efficiency of deletion of SREBP1 or SREBP2 in mHCC cells was confirmed by immunoblotting. Vinculin is shown as loading control.

Supplementary Figure S6: Related to Figure 6

a) Diagram depicting the generation of LSCC tumours in mice using intra-tracheal delivery of adeno-associated virus particles containing expression cassettes coding for sgRNAs targeting *Kras* and *p53* plus a template for homologous recombination to introduce a *Kras* oncogenic mutation (HR-G12D). These elements were transduced either alone (KP) or in combination with gRNAs targeting Cas9 to the *Lkb1* (KPL), *Usp28* (KPLU) of *Srebf2* (KPLS2) loci.

b) Lung tissue from KP mice was stained for the adenocarcinoma marker thyroid transcription factor 1/NK2-homeobox 1 (Ttf-1/NKX2-1) and Hmgcs1 by immunohistochemistry. Haematoxylin and eosin (H&E) staining is also shown.

c) Boxplot showing quantification of Hmgcs1 staining in non-transformed tissue (NT) and tumour tissue (T) from KP mice. Staining intensity of cytoplasmic staining is shown. (n=3 individual tumours; NT: n=3,933 cells; T: n=3,811 cells; ***p<0.001, Mann-Whitney test).

d) Lung tissue from KPL mice was stained for Ttf-1, the squamous marker keratin 5 (Krt5), Usp28 and Hmgcs1 by immunohistochemistry. Haematoxylin and eosin (H&E) staining is also shown.

e) Boxplots showing quantification of Hmgcs1 and Usp28 staining in non-transformed tissue (NT) and tumour tissue (T) from KPL mice. Staining intensity and % positive cells of cytoplasmic staining for Hmgcs1 and nuclear staining for Usp28 is shown. (Usp28: n=7 individual tumours; T: n=15,199 and 35,010 cells; Hmgcs1: n=12 individual tumours; T: n=22,168 and 44,492 cells; ***p<0.001, Mann-Whitney test).

f) Tissue sections from KPL tumours were stained for Ttf-1, Krt5, Usp28 and Hmgcs1 by immunohistochemistry. Haematoxylin and eosin (H&E) staining is also shown.

g) Boxplot showing quantification of Hmgcs1 staining in non-transformed lung tissue (NT), lung adenocarcinoma (ADC) and lung squamous cell carcinoma (SCC) tumours in KPL and mice. Staining intensity of cytoplasmic staining is shown. (n=3 individual tumours; NT:

n=12,825 cells; KPL-LADC: n=6,825 cells; KPL-LSCC= 7,040 cells; ***p<0.001, one-way ANOVA with post-hoc Tukey test).

h) Lysates from KPL and KPLU tumours were analysed for the expression of Usp28, Hmgcs1 and Fdft1 by immunoblotting. Vinculin is shown as a loading control.

i) Ratio of tumour area relative to total lung area in KPL and KPLS1 mice. Data are displayed as mean ± SD (KPL: n = 11; KPLS1: n = 13; Mann-Whitney test).

Supplementary Figure S7: Related to Figure 7

a) A431, H2170 and LUDLU cells were treated with indicated concentrations of simvastatin (Sim) or AZ1 in medium containing 10% FCS for 72 hours. Drug synergy was calculated using ZIP model.

a

Gene Symbol	log 2FC	p-value	q-value
ACAT2	-0.35	**0.008**	**0.023**
HMGCS1	-1.99	**0.000**	**0.001**
HMGCR	-0.61	0.082	0.136
MVK	-0.37	**0.016**	**0.039**
PMVK	-0.64	**0.001**	**0.007**
MVD	-0.58	**0.007**	**0.022**
IDI1	-0.35	**0.005**	**0.017**
FDPS	-1.05	**0.009**	**0.023**
FDFT1	-0.67	**0.001**	**0.007**
SQLE	-0.71	**0.002**	**0.009**
LSS	0.65	**0.001**	**0.008**
CYP51A1	-0.42	**0.028**	0.059
LBR	0.17	0.338	0.427
MSMO1	-0.58	**0.033**	0.066
NSDHL	0.35	**0.018**	**0.043**
HSD17B7	-0.04	0.895	0.923
EBP	0.66	0.145	0.215
DHCR7	0.16	0.181	0.258
DHCR24	0.23	**0.005**	**0.017**
GGPS1	-0.93	**0.000**	**0.002**
FNTA	-0.57	0.204	0.284
PGGT1B	-0.25	0.159	0.231

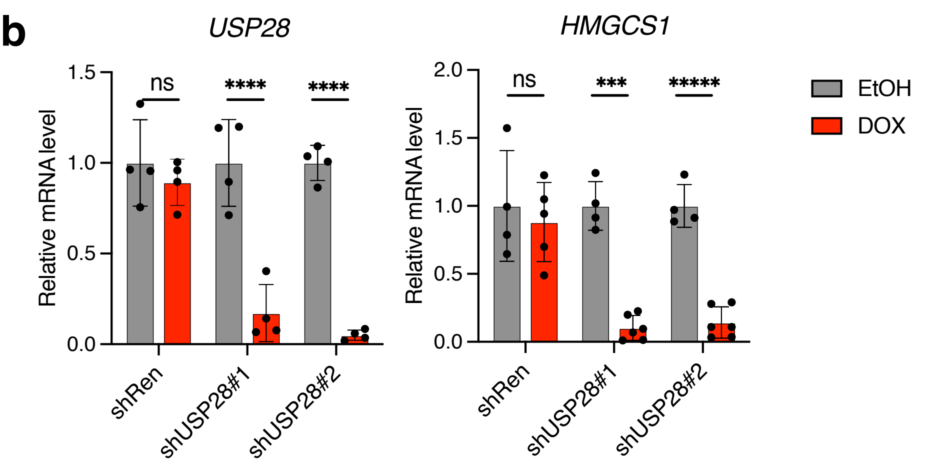

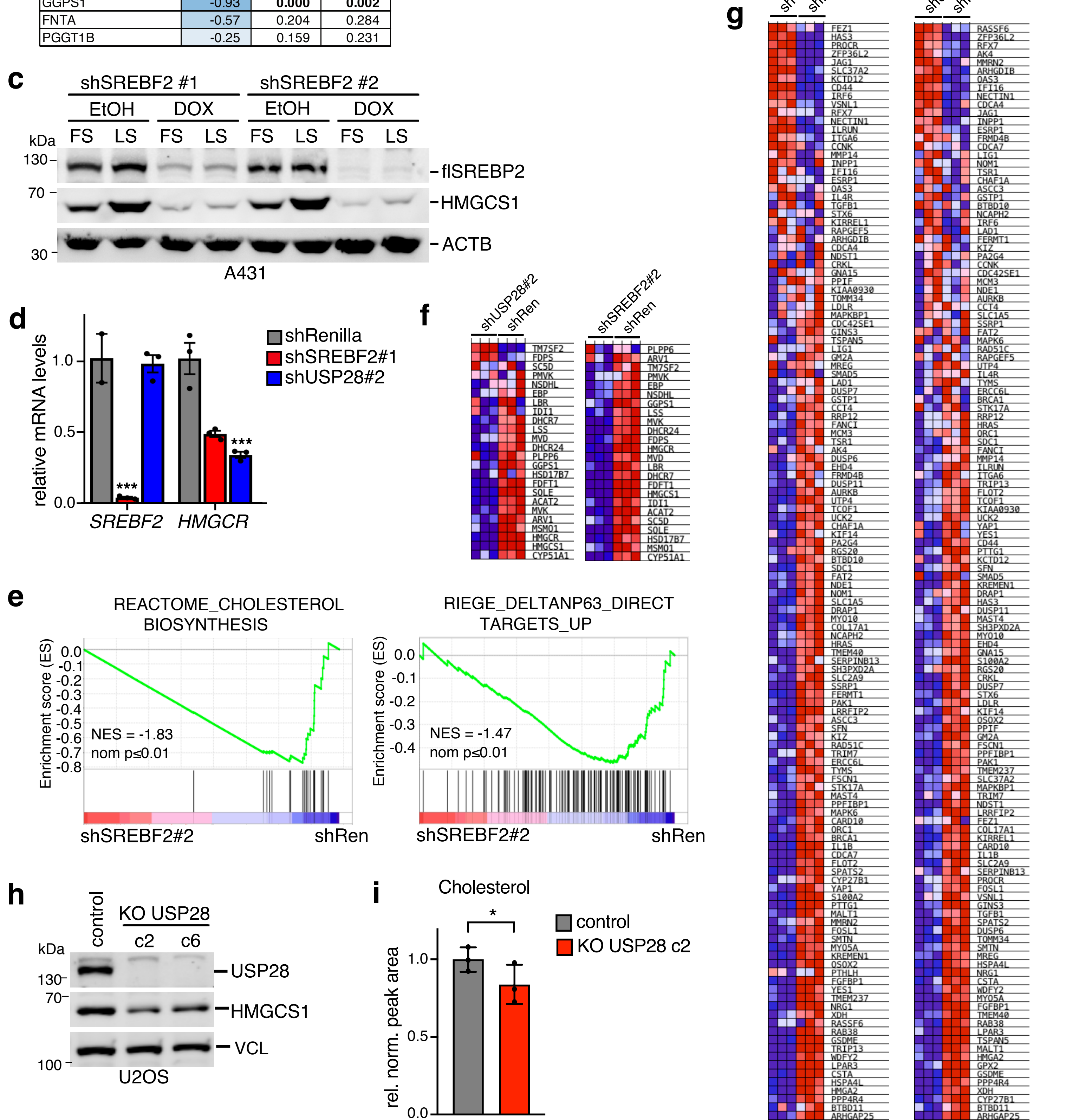

Maier et al. Figure S1

a

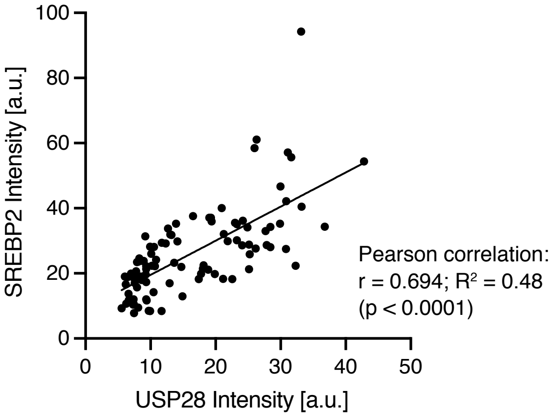

Maier et al. Figure S2

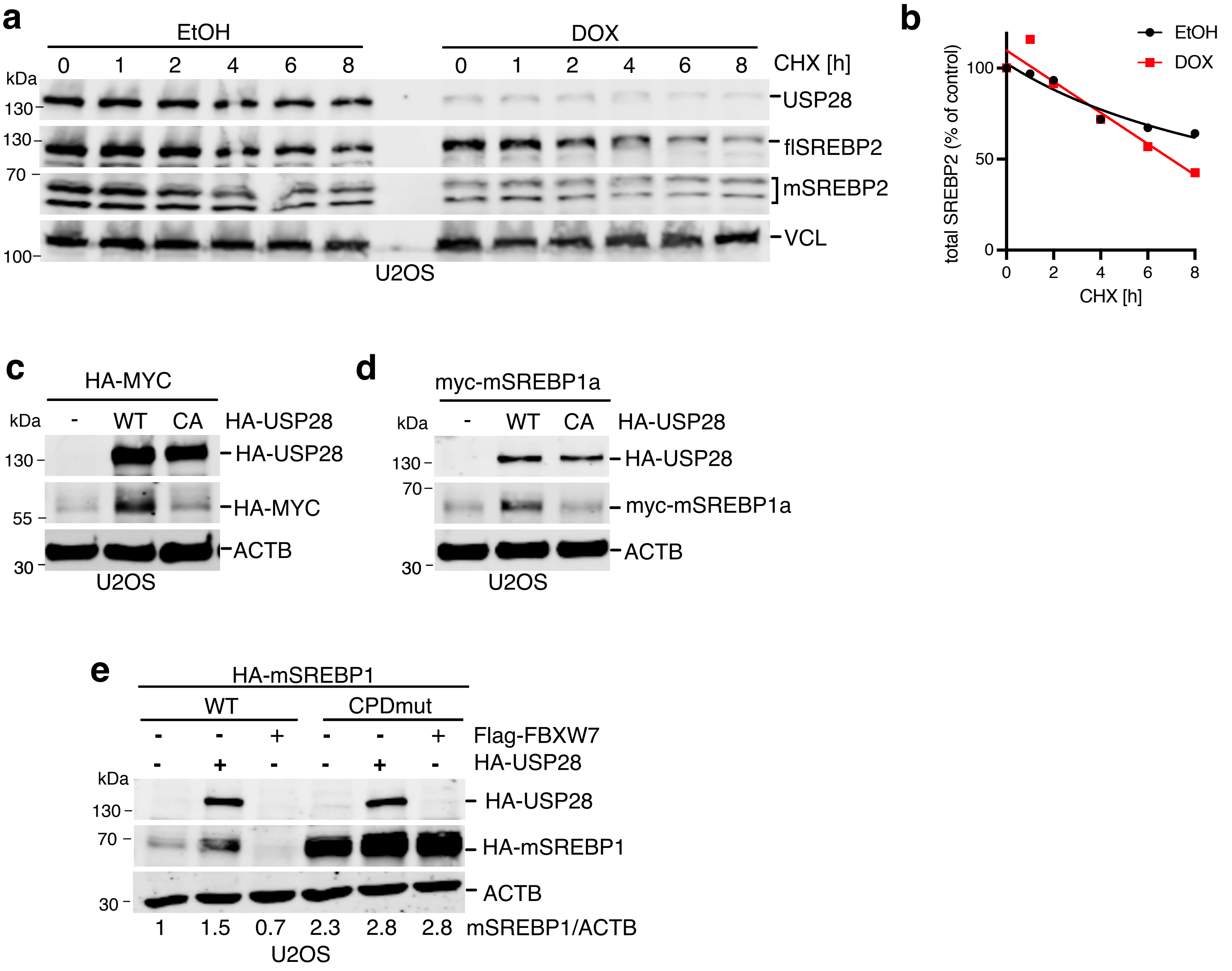

Maier et al. Figure S3

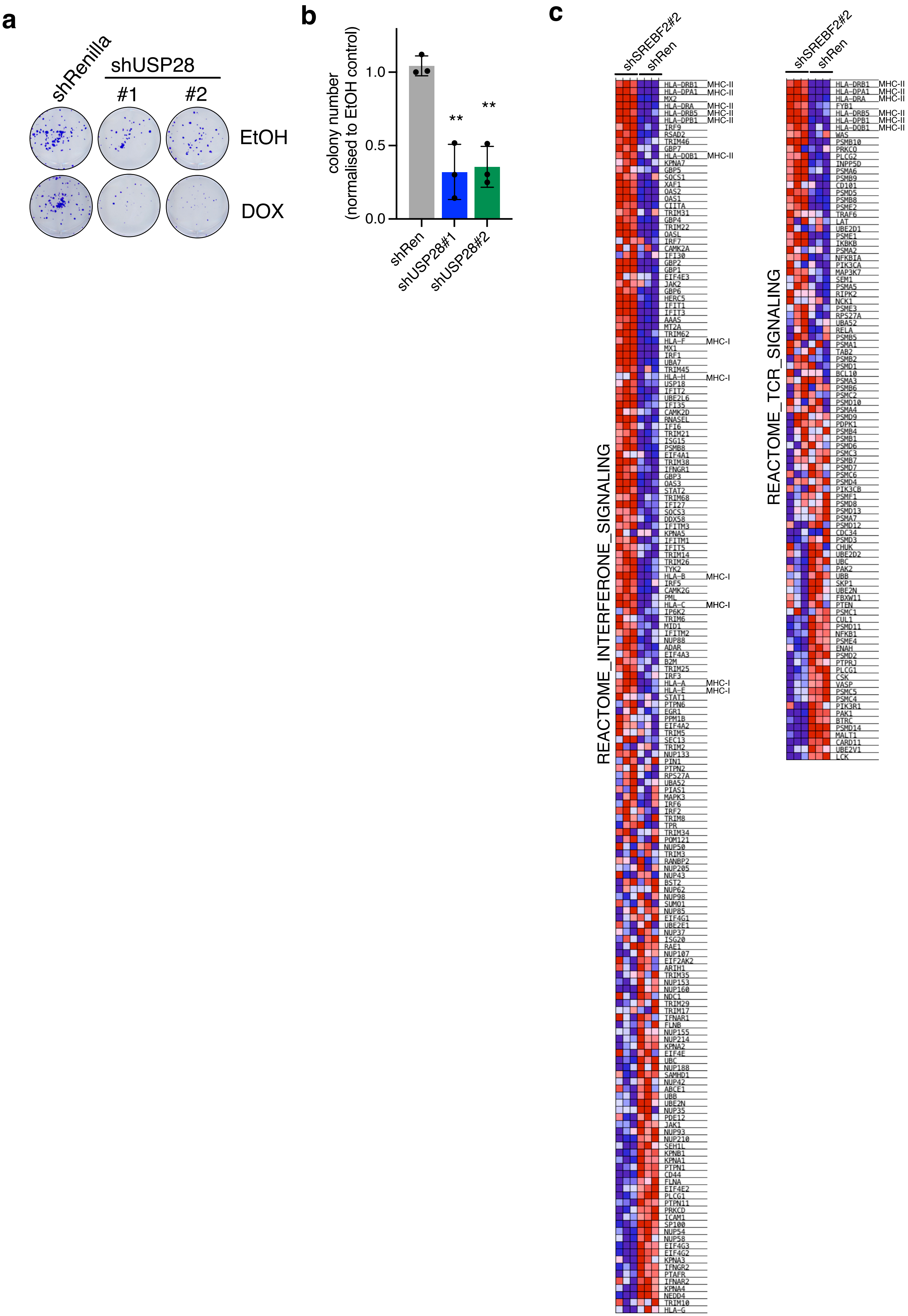

Maier et al. Figure S4

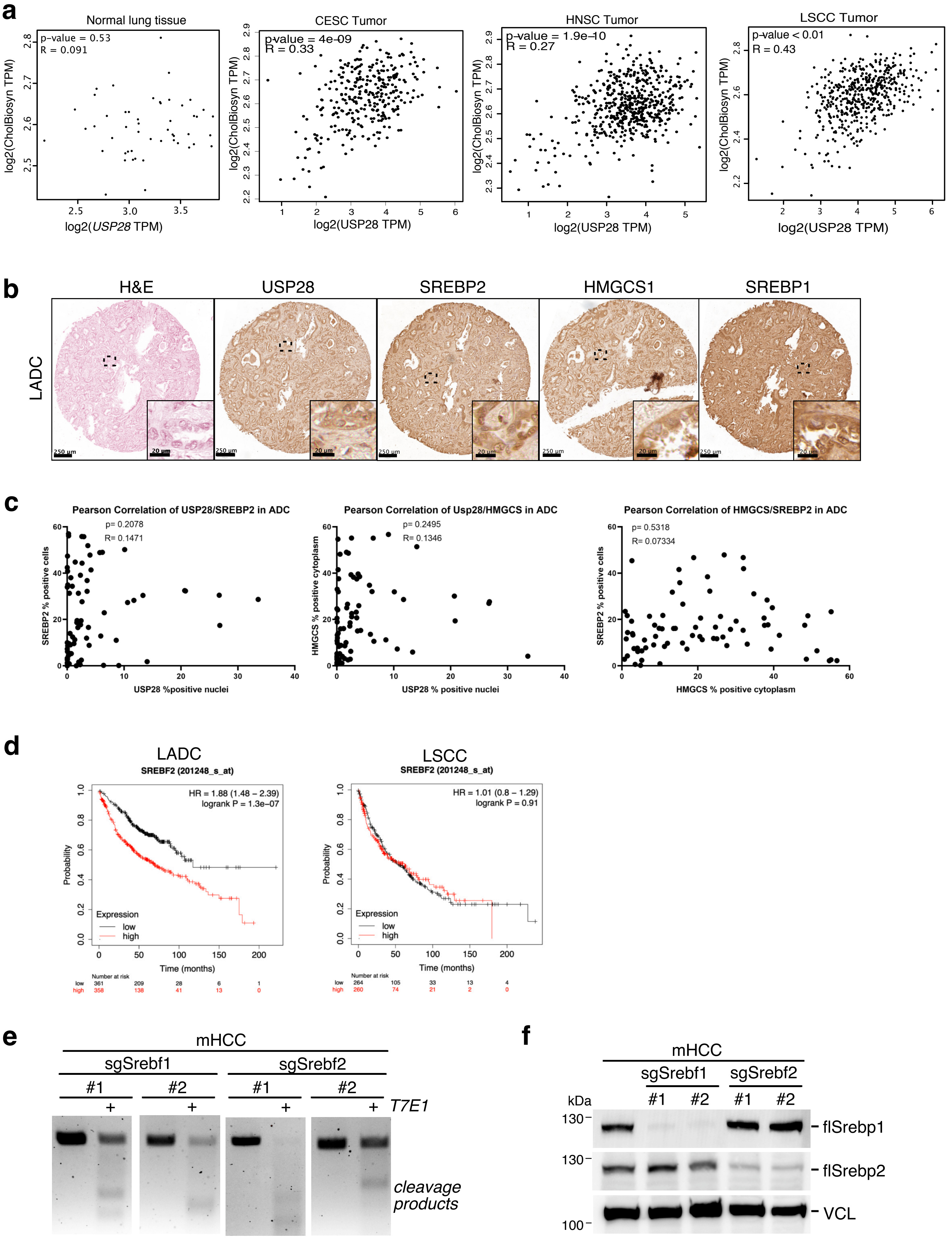

Maier et al. Figure S5

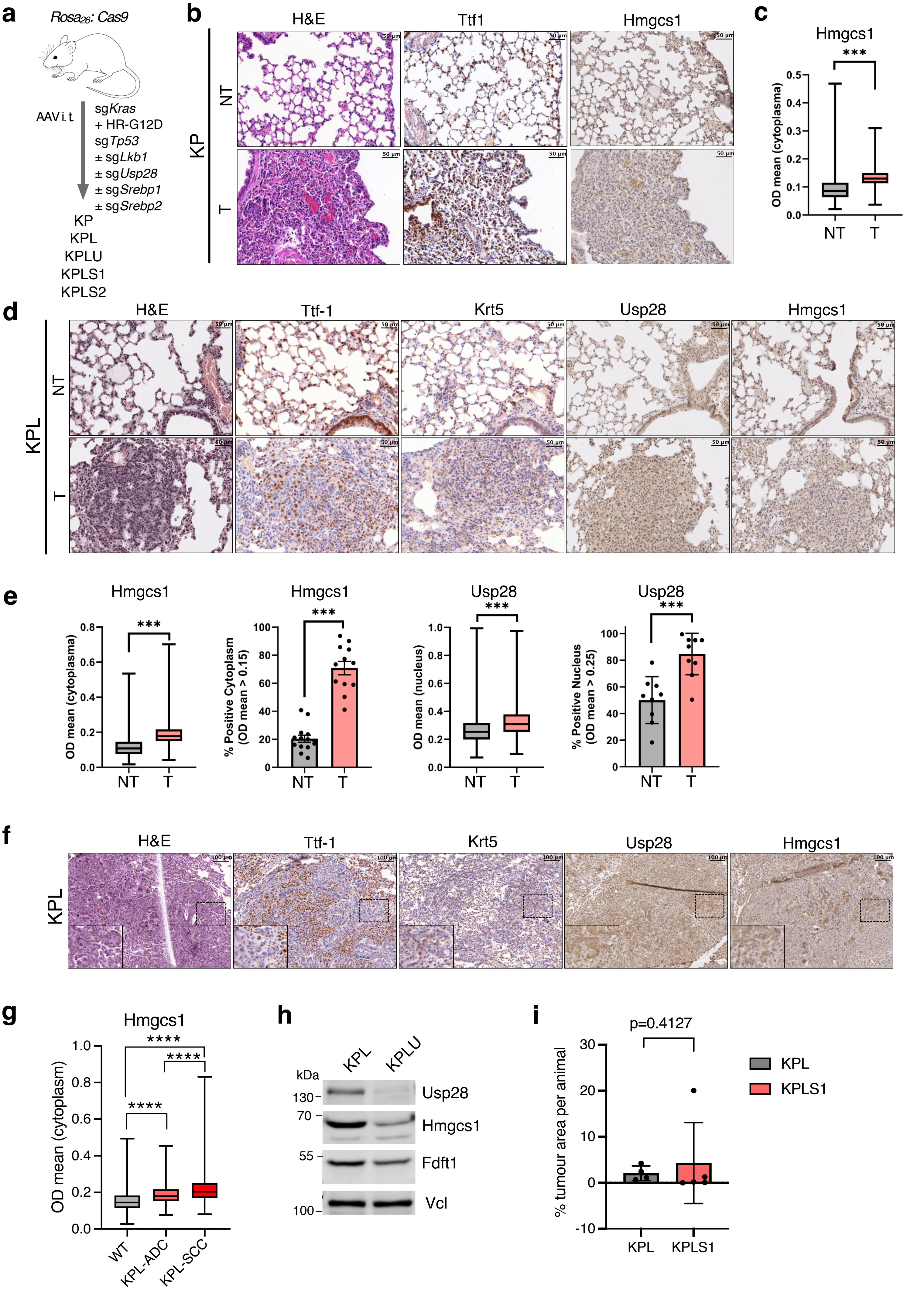

Maier et al. Figure S6

a

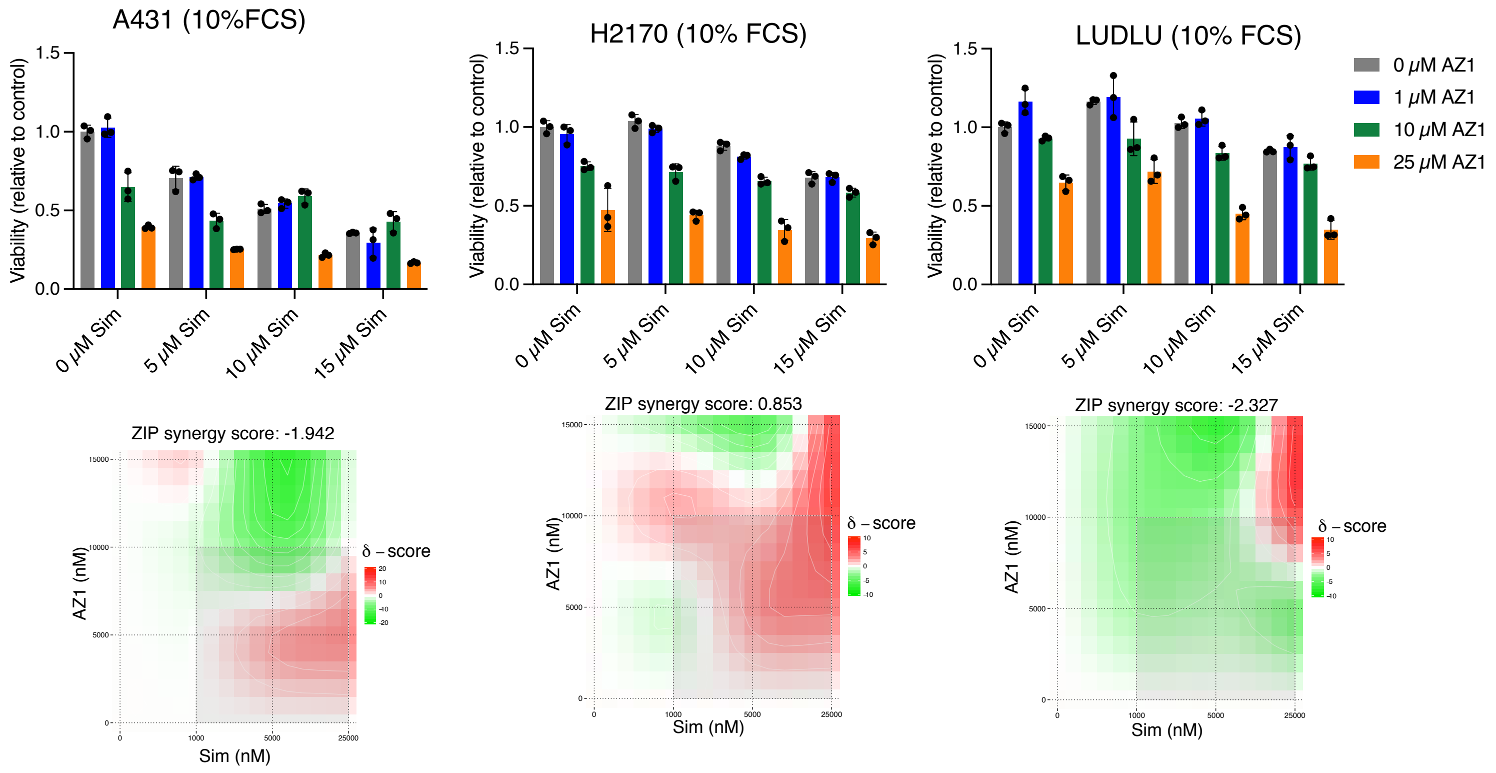

Maier et al. Figure S7
