# Supplementary material for: USP28 controls SREBP2 and the mevalonate pathway to drive tumour growth in squamous cancer
Source: Cell Death Differ. 2023 May 18;30(7):1710–25. doi: 10.1038/s41418-023-01173-6 (PMC10307777; doi:10.1038/s41418-023-01173-6)

Figure 1a:

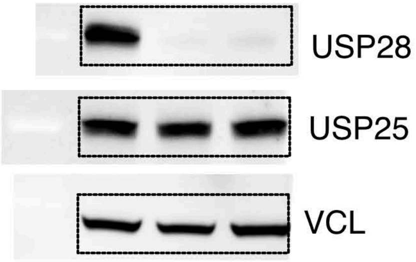

Figure 1f:

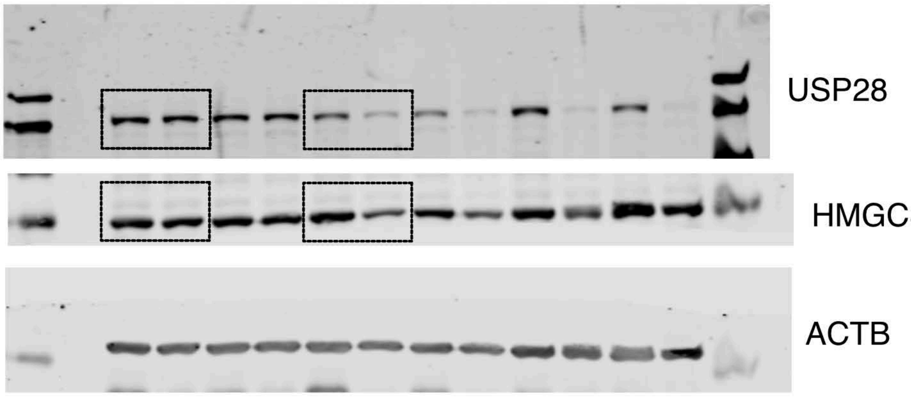

Figure S1c

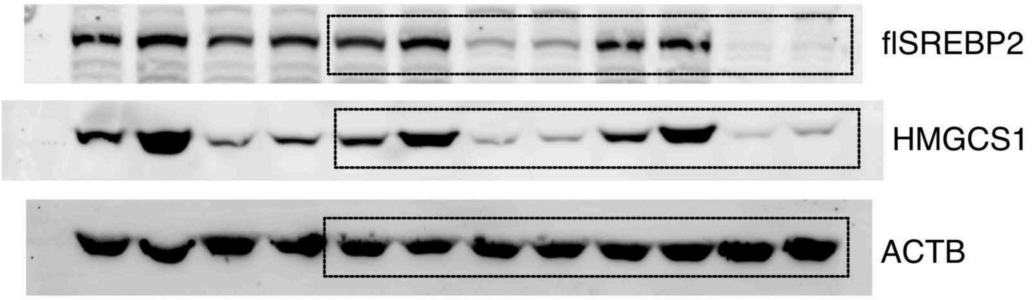

Figure S1h

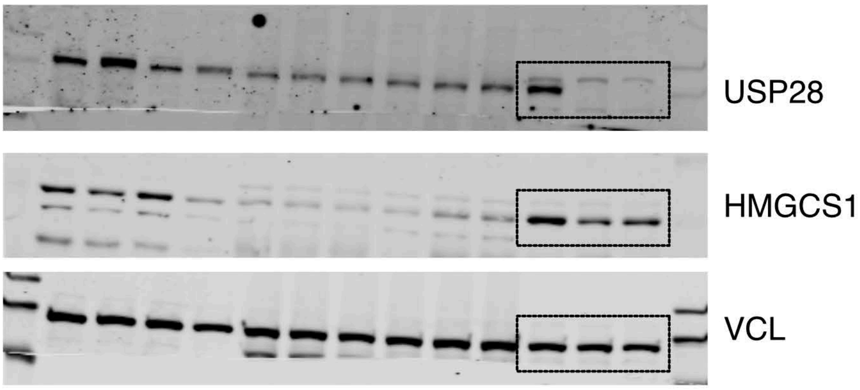

Figure 2a:

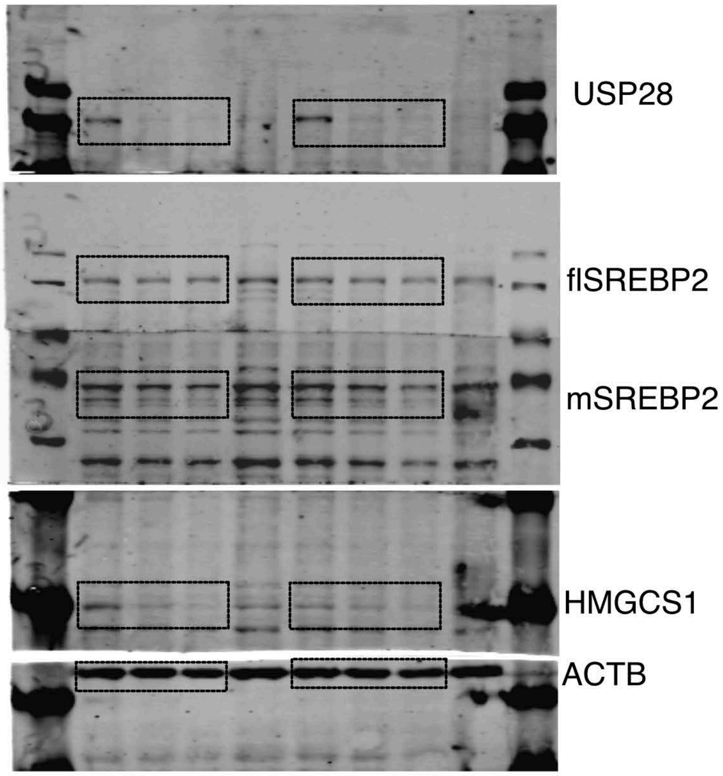

Figure 2c:

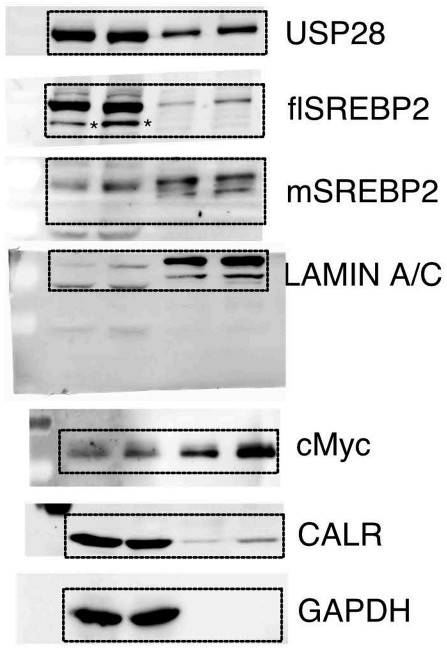

Figure 2e

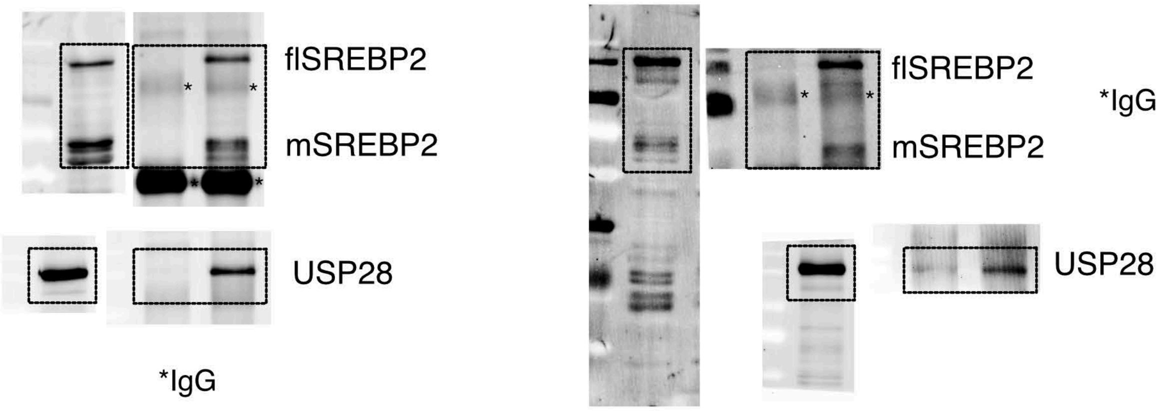

Figure 2f

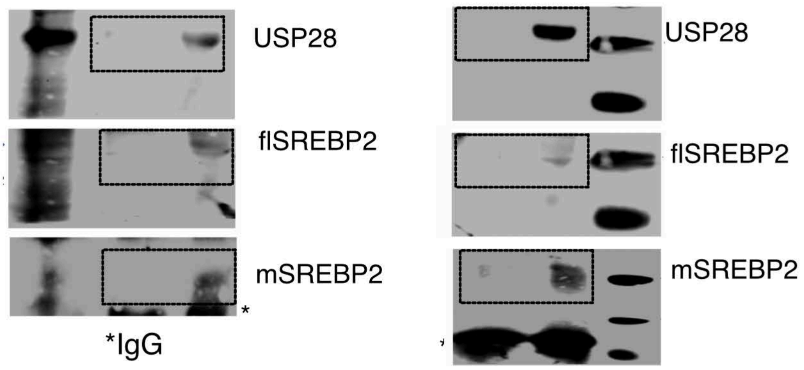

Figure 3a:

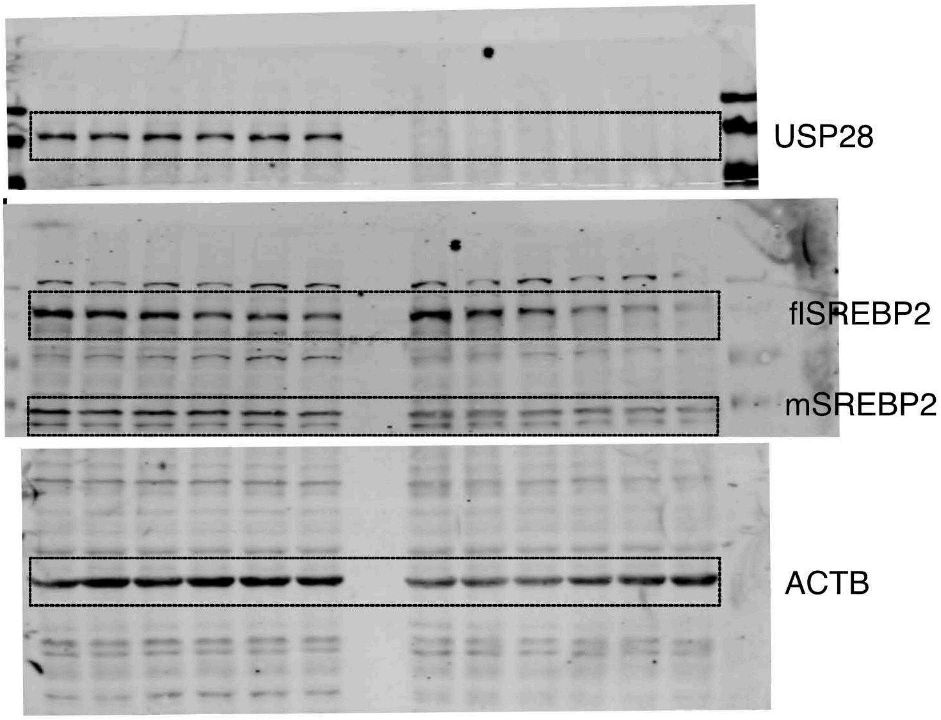

Figure 3f:

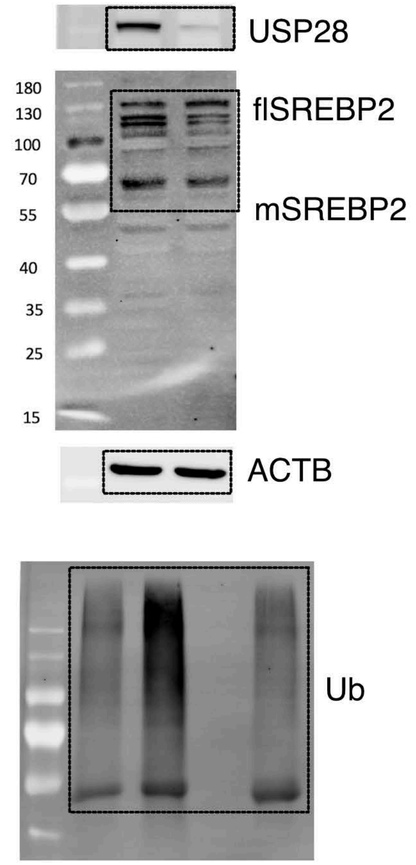

Figure 3c:

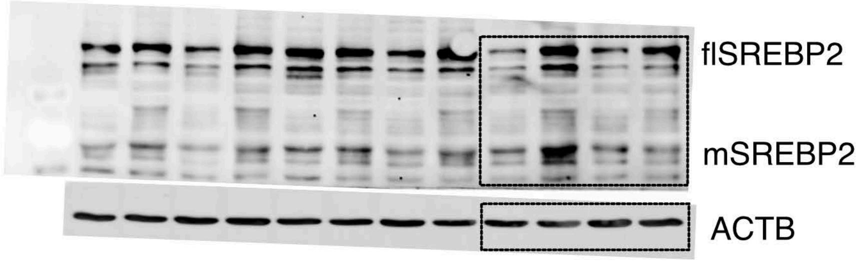

Figure 3d:

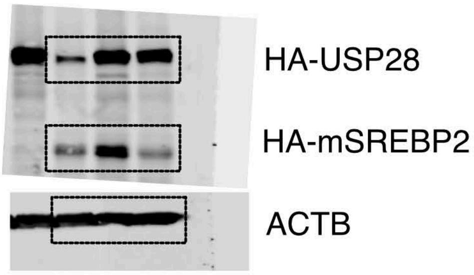

Figure 3e:

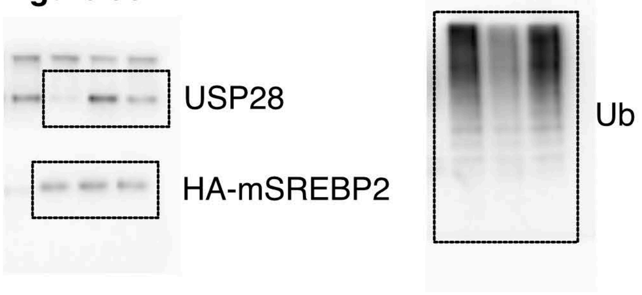

Figure 3g:

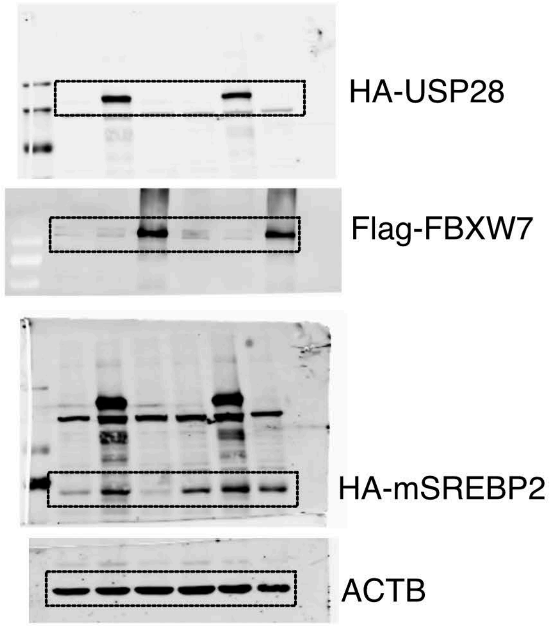

Figure S3a:

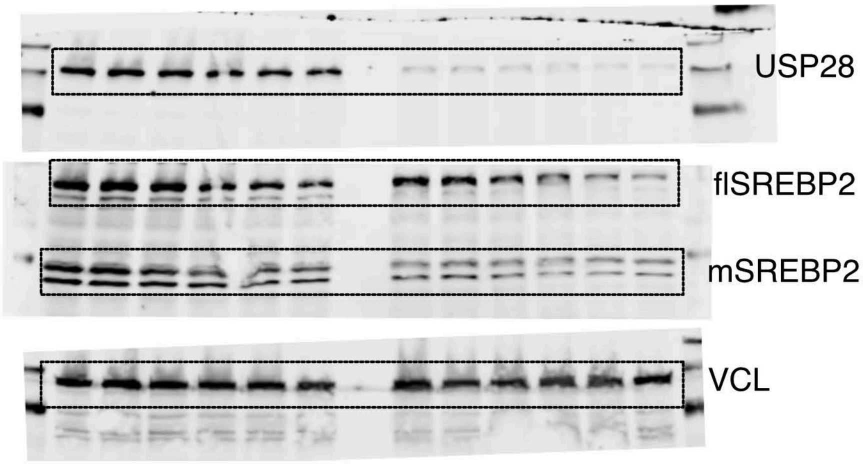

Figure S3c:

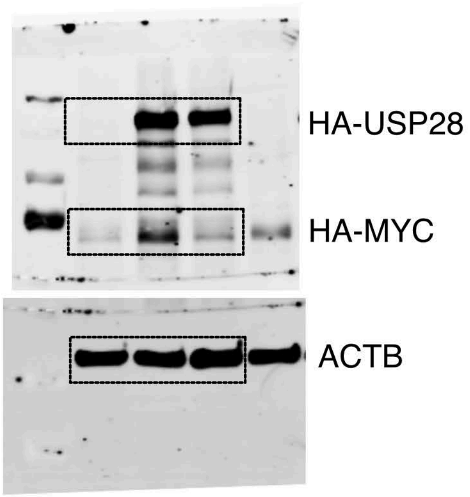

Figure S3d:

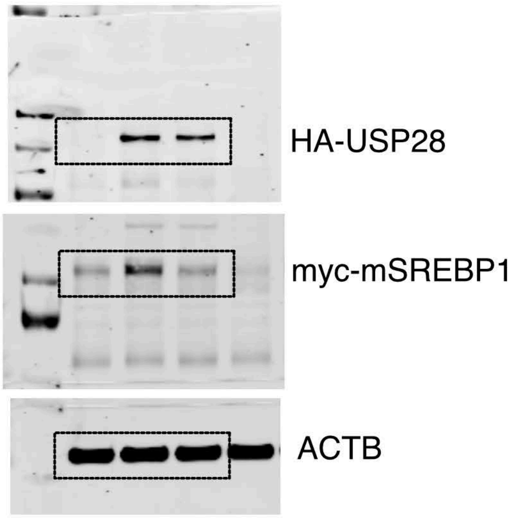

Figure S3e:

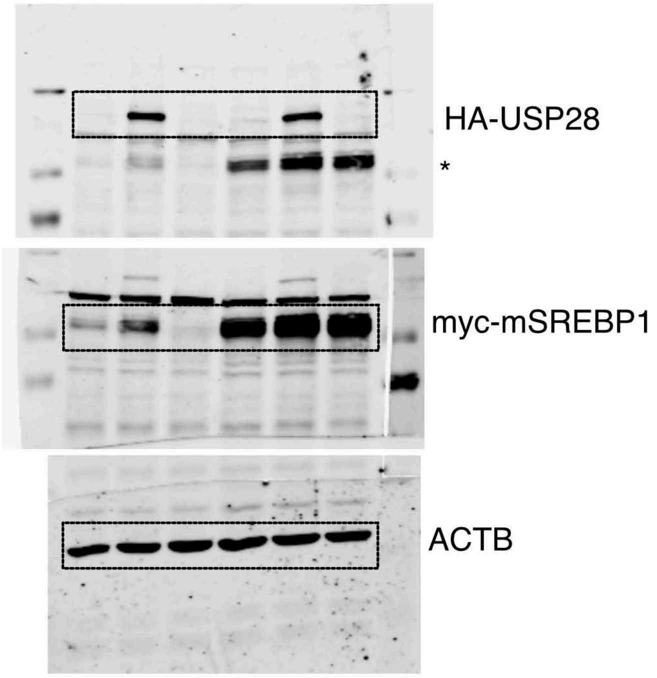

*previous antibody signal

Figure S5e:

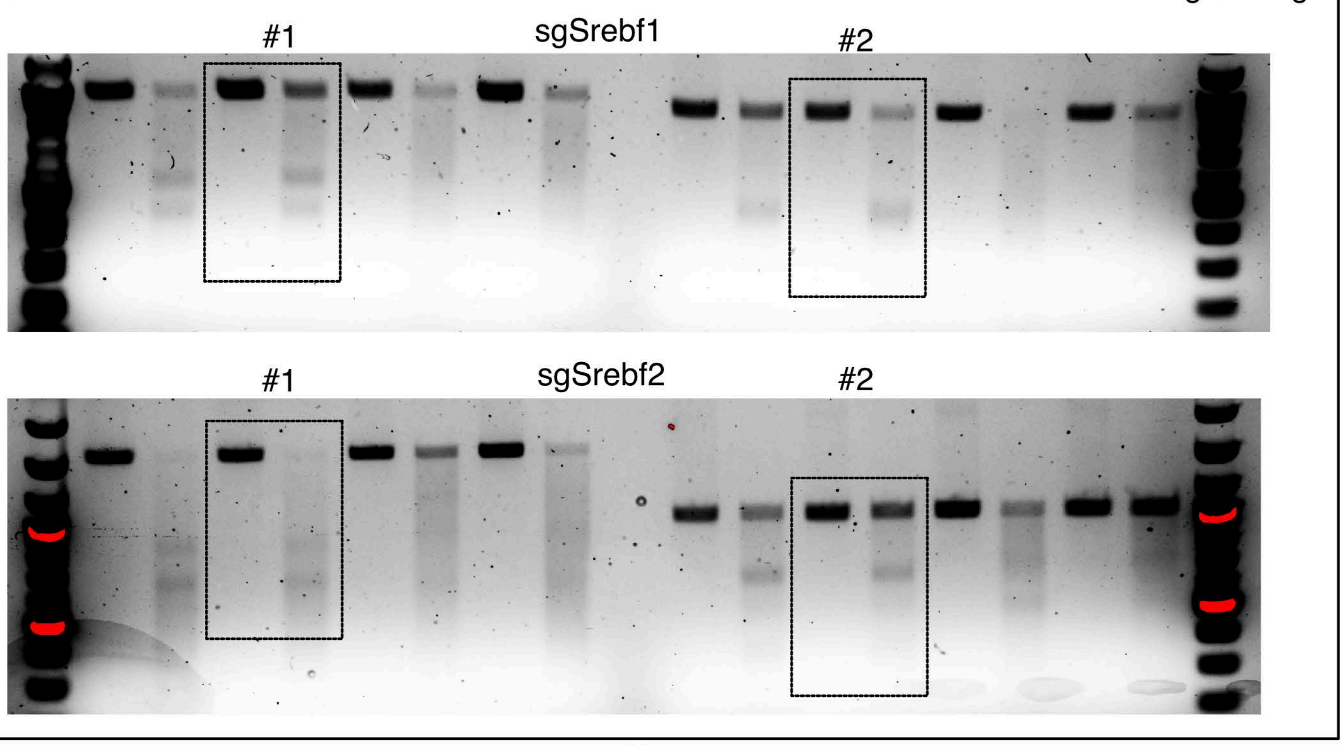

Figure S5f:

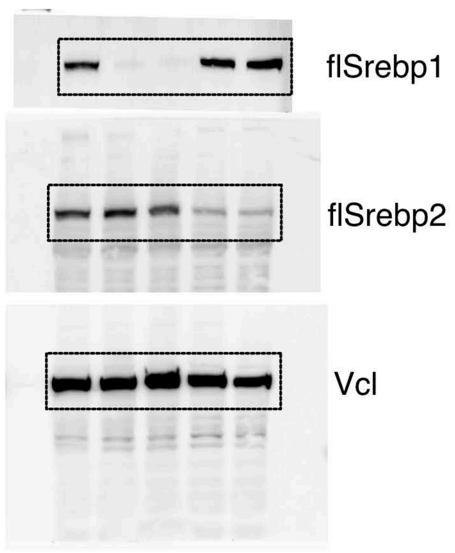

Figure S6h:

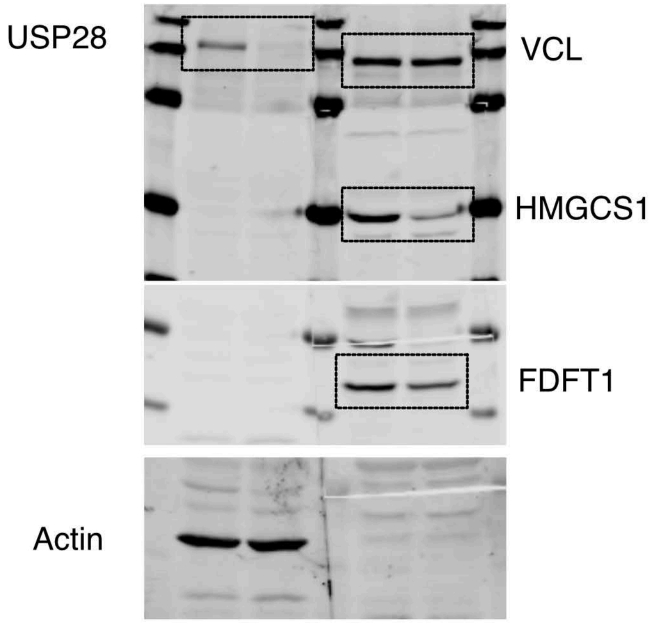

Supplement: Supplementary file 2 — Uncropped WB images [file 41418_2023_1173_MOESM2_ESM.pdf]
